# Chinese olive (*Canarium album* Rauesch.): a critical review on its nutritional value, phytochemical composition, health benefits, and practical applications

**DOI:** 10.3389/fphar.2023.1275113

**Published:** 2023-11-29

**Authors:** Kuo Yu, Yan Wang, Wen-Jing Hu, Zhao-Jiong Zhang, Guan-Yu Zhou, Shi Sun, Hai-Xue Kuang, Meng Wang

**Affiliations:** ^1^ Beidahuang Industry Group General Hospital, Harbin, China; ^2^ Key Laboratory of Basic and Application Research of Beiyao, Ministry of Education, Heilongjiang University of Chinese Medicine, Harbin, China

**Keywords:** Chinese olive, nutritional value, phytochemical composition, health benefits, practical applications

## Abstract

Chinese olive is a popular fruit with a long history of cultivation and consumption. As a fruit with edible, nutritional, and even medicinal value, the Chinese olive has attracted increased interest from both nutrition researchers and health-conscious consumers. Chinese olive is a rich nutrient source, including essential and non-essential amino acids, various fatty acids, organic acids, vitamins, microelements, and high-quality dietary fibers. It is also an important natural source of phytochemicals such as phenolic acids, flavonoids, phenylpropanoids, and other bioactive compounds. The nutritional and phytochemical compounds obtained from the Chinese olive exhibit unique and potent biological activities, explaining its various benefits to human health, including anti-*Helicobacter pylori*, anti-influenza, anti-diabetes, anti-inflammatory, anti-tumor effects, among others. This review focuses on recent studies on Chinese olives and aims to summarize the major advances in their nutritional value, phytochemical composition, health benefits, and practical applications. It provides a reference for further research on Chinese olives and their properties and the development of novel functional products.

## 1 Introduction

**FIGURE 1:**
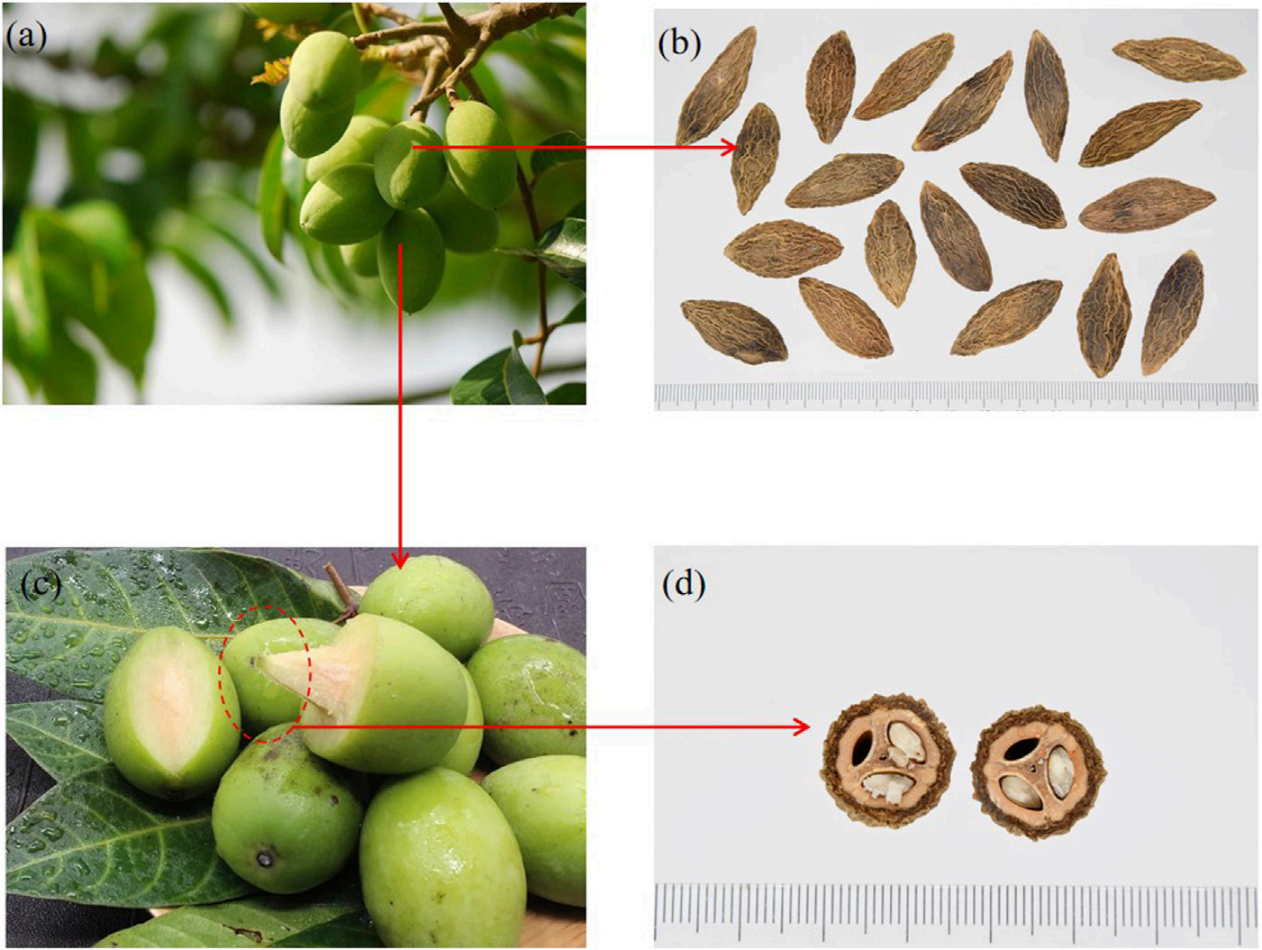
Plant images of Chinese olive. **(A)** The whole plant. **(B)** Dried fruit. **(C)** Fresh fruit. **(D)** Cross section of kernel. (Images from network and public sources).

Nature provides abundant food for human consumption, and the different food sources and types can be divided into animal and plant sources (Comerford et al., 2021; McClements and Grossmann, 2021). Food products of animal origin are mainly a source of proteins and fats, and those of plant origin are more often a rich source of nutrients such as vitamins, carbohydrates, and bioactive molecules. Among them, fruits are very beneficial and an indispensable food in our daily diet (Wallace et al., 2020; Wang et al., 2022a). Chinese olive, the mature fruit of *Canarium album* (Lour.) Rauesch., which belongs to the *Burseraceae* family, is a famous tropical and subtropical fruit (Chen et al., 2018). It originates from South China with a long cultivation history and consumption spanning more than 2,000 years. Chinese olive grows in low-altitude coastal forests, mountain slopes, and low hilly areas and is currently cultivated mainly in only small areas, mainly in the wild (Ye et al., 2021). China is the largest producing area of Chinese olive, especially Fujian Province, its main origin, with over 140,000 acres of planting area. In addition to China, Chinese olive is cultivated in Vietnam, Japan, Laos, Myanmar, Thailand, Philippines, Malaysia, Indonesia, India, and Sri Lanka, encompassing almost the entire of Asia (Figure 1) (Jia et al., 2016; Fan et al., 2022). Chinese olive is a widely popular fruit as well as a nutritious food popular among health-conscious consumers. Thus, it has attracted increased interest from researchers in the field of human nutrition and health-conscious consumers (Yu et al., 2021).

Chinese olives are different from olive oil (*Olea europaea* L.). The main purpose of consuming Chinese olives is to taste their flesh and absorb nutrients, rather than squeezing oil. Chinese olive, as an edible fruit, has a unique and memorable taste. Its peel changes from green to yellow-green during the ripening process. After the fruit ripens, the main edible part is its pulp, which has delicate aromas and a sour taste and creates a lasting aftertaste (Lai et al., 2021). The Chinese olive kernel is spindle-shaped with longitudinal cracks, and there are seeds in each of the three chambers, rich in oil that can be used for edible oil extraction (Mei et al., 2017). As a fresh food fruit, the Chinese olive has rich nutritional value. Studies have shown that it is rich in vitamins and can contain 10 times higher vitamin C (VC) than apple and 5 times higher than pear and peach (Toumi et al., 2022). It is rich in the trace element selenium, also known as the “life element” helping the organism retard aging processes (Mojadadi et al., 2021). Chinese olive contains various essential amino acids. Moreover, it is rich in plant-derived calcium with iron, which can be easily absorbed by the body (He and Xia, 2007a). Except for being consumed fresh, Chinese olive is also part of the human diet in many other forms. It can be soaked in water to be used as a fruit tea, a condiment for stew and soup, and a raw material to make jam together with various fruits. It can also be processed into preserves, candies, leisure snacks, and even used as fermentation raw material for winemaking (Wang et al., 2019).

Furthermore, the Chinese olive is a rich source of high-quality dietary fibers, vitamins, microelements, carbohydrates, and other nutrient-rich substances (Lai et al., 2019). It has attracted the scientific community’s attention as an important plant with edible, nutritional, and even economic value. Chinese olive has been fully utilized and deeply developed during its long-term consumption and application history. It is not only served as delicious food but also as effective empirical medicine. It is worth noting that the Chinese olive was among the first to be included in the Chinese pharmacopeia as a fruit. It is also included in the current list of “affinal drugs and diet” in China issued by the National Health Commission. Thus, the health benefits of Chinese olives have been fully recognized. It is used as a “affinal drugs and diet” as well as nutritional food, functional food, and an important bioactive ingredient source for traditional oriental medicines (Xiao et al., 2022a). The realization of Chinese olive benefits to human health was initially through observation and experience. After Chinese olive consumption can increase saliva secretion and eliminate the thirst sensation. Additionally, it can alleviate the swelling and pain of the throat and reduce cough and phlegm (Cheng et al., 2016). Furthermore, it has been occasionally observed that eating Chinese olives can eliminate food poisoning caused by excessive or wrong consumption of fish and crabs (He and Xia, 2011). In modern times, it is mainly scientific research that has uncovered the potential effects of Chinese olive on human health. For example, Chinese olive can effectively control *Helicobacter pylori* to reduce the risk of stomach diseases and control the influenza virus to fight influenza effectively (Xiao et al., 2022b). It possesses anti-diabetic properties to improve the body’s metabolic functions. Therefore, Chinese olive’s nutritional and medicinal value is widely recognized in line with consumer demand for a healthy diet, adequate nutritional intake, and dietary therapeutic properties.

In recent years, many studies have been carried out worldwide on the Chinese olive across different fields. It is generally believed that the Chinese olive serves as nutritious fruit or natural functional food and is gaining immense popularity in daily diet due to its vital plant metabolites such as polyphenols, flavonoids, triterpenes, coumarins, polysaccharides, and other bioactive compounds (Wang et al., 2022b). These phytochemicals obtained from the Chinese olive exhibited unique and potent biological activities, which can explain various benefits of Chinese olive to human health, including anti-*Helicobacter pylori*, anti-influenza, anti-diabetes, anti-tumor effects, and others (Guo et al., 2008; Kong et al., 2018). Due to this, it has attracted increasing research interest, which provides promising prospects for its future applications as a food and nutritional source.

Nowadays, people want fresh food and rich nutrition and health benefits while consuming delicious food. The rich nutrients in Chinese olives and the bioactive phytochemicals from natural sources, which provide various benefits to human health, meet people’s demand for functional food and nutritional supplements. To this end, there are currently no literature review resources on Chinese olive to the best of our knowledge. At the same time, the research on Chinese olive has gradually increased and has become more complex and sophisticated. To avoid duplication and confusion of research work, there is an urgent need to sort out the existing research on Chinese olive. In this paper, the nutritional and phytochemical composition, as well as the health benefits of Chinese olives are reviewed. The current and potential applications are summarized, which can provide a reference and basis for further applied research.

## 2 Nutritional value

Protein, fat, and carbohydrate are the three calorigenic nutrients (Großkopf and Simm, 2020). Chinese olive is a high-content fruit in terms of the above compounds, containing 29.5 g of protein, 52.8 g of fat, and 0.2 g of carbohydrate per 100 g fresh kernel. Meanwhile, At the same time, Chinese olives are rich in amino acids, fatty acids, organic acids, vitamins (C, B1, B2), retinol, carotene, and other nutrients, as well as various trace elements such as calcium, iron, and phosphorus.

The content of mineral elements, such as potassium (587 mg/100 g), calcium (226.2 mg/100 g), and magnesium (186 mg/100 g) in fresh Chinese olives, is relatively high (Table 1). The mineral elements from Chinese olives are more easily absorbed and utilized by the human body, and their absorption rate is far higher compared to synthetic elements (He and Xia, 2007b).

**TABLE 1:** Nutrition of Chinese olive.

Items	Content (/per 100 g) (kernel)
Protein	29.5 ± 0.9 g
Crude fat	52.8 ± 0.4 g
Ash	4.9 ± 0.08 g
Total carbohydrate	0.2 ± 0.03 g
Moisture	5.2 ± 0.1 g
K	587 ± 3.4 mg
Ca	226 ± 1.8 mg
Na	40.1 ± 1.4 mg
Mg	186 ± 2.7 mg
Fe	12.4 ± 0.3 mg
Mn	2.5 ± 0.4 mg
Zn	1.8 ± 0.2 mg
Cu	0.9 ± 0.1 mg

Proteins are the material basis of life, being a part of all metabolic activities (Boukid et al., 2022). Amino acids are the protein building blocks (Xiao and Guo, 2022). The World Food and Agriculture Organization (FAO) and World Health Organization (WHO) suggest that the ideal protein content and the ratio of essential amino acids to non-essential amino acids in protein should be 40% and 0.6%, respectively (Romano et al., 2019). Chinese olive contains 18 different amino acids, including 8 essential amino acids (threonine, valine, methionine, isoleucine, leucine, phenylalanine, lysine, tryptophan) and 10 non-essential amino acids (aspartic acid, serine, glutamic acid, glycine, alanine, tyrosine, histidine, arginine, proline, cystine). The essential amino acids content and the ratio of essential amino acids to non-essential amino acids are very close to the ideal reference protein model values suggested by FAO and WHO (Table 2). This suggests that Chinese olive is an ideal and appropriate daily amino acid supplement source as fresh fruit.

**TABLE 2:** Amio acid composition in Chinese olive.

Amino acid	Content (g/100g)	
	Flesh	Kernel
Aspartic acid (Asp)	0.20	2.47
Threonine (Thr)	0.07	0.83
Serine (Ser)	0.10	1.10
Glutamicacid (Glu)	0.49	5.02
Glycine (Gly)	0.04	1.25
Alanine (Ala)	0.02	0.96
Valine (Val)	0.07	1.30
Methionine (Met)	0.02	0.71
Isoleucine (Ile)	0.08	1.05
Leucine (Leu)	0.16	1.87
Tyrosine (Tyr)	0.03	0.83
Phenylalanine (Phe)	0.11	1.24
Lysine (Lys)	0.20	0.72
Histidine (His)	0.02	0.61
Arginine (Arg)	0.15	3.19
Proline (Pro)	0.06	1.16
Tryptophan (Trp)	0.05	1.18
Cystine (Cys)	0.03	0.52

Chinese olive fresh kernel is rich in fatty acids, with 13 fatty acids detected, among which unsaturated fatty acids amount up to 73.3%. The most abundant fatty acids are linoleic acid (41.8%), oleic acid (30.5%), palmitic acid (18.0%), stearic acid (7.8%), and linoleic acid as the main components. Linoleic acid is an essential fatty acid for humans, and it can combine with cholesterol to reduce the cholesterol content in the blood, having a beneficial effect on human health (Marangoni et al., 2020). Thus, edible oil from the Chinese olive kernel is an excellent plant nutritional source.

Chinese olive contains many types of organic acids, which provide a pleasant taste and a long aftertaste. Organic acids are overall important flavor substances in fruits, providing a rich taste (Liu et al., 2022). The organic acids in Chinese olive mainly include malic acid, citric acid, tartaric acid, quinic acid, oxalic acid, and a small amount of fumaric acid and acetic acid. Malic acid is the main organic acid in Chinese olives, with a content of 463.6–766.1 mg/100 g Fresh weight (FW). Foods of plant origin can produce rich small molecular compounds during photosynthesis and metabolism, and these chemical compounds possess potential beneficial biological activities (Oldroyd and Leyser, 2020). Chinese olive contains many types of phytochemical compounds, which are potentially responsible for its beneficial properties, which are evident after the consumption of Chinese olives.

## 3 Phytochemical composition

Chinese olives are rich in nutrients and contain many plant metabolites, including phenolic acids, flavonoid, phenylpropanoid, triterpenoid, and other bioactive compound, among others (Table 3). Phenolic compounds are substances containing polyphenol functional groups in their molecular structures. They are mainly abundant in plant sources, such as vegetables and fruits. As a nutrient-rich fruit, the type and content of the phenolic compound account for the largest proportion of chemical components in Chinese olives. 100 g of fresh Chinese olives contains 1174.0–1799.6 mg of phenolic compounds. Phenolic compounds are generally considered the main chemical compounds responsible for Chinese olives’ pharmacological and therapeutic effects and are also responsible for the fruit’s bitter taste. Phenolic compounds in Chinese olives are further subdivided into phenolic acids, flavonoids, and phenylpropanoids.

**TABLE 3:** Major polyphenol, flavonoid, and phenylpropanoids compounds in Chinese olive.

Type	Name	Molecular formula	Extract solvent	References
Polyphenol	Brevifolin (1)	C_12_H_8_O_6_	Acidic ethylacetate	Ito et al. (1990)
	Brevifolin carboxylic acid (2)	C_13_H_8_O_8_	80% acetone	He and Xia (2007a)
	Chebulagic acid (3)	C_41_H_30_O_27_	Water	Chang et al. (2017)
	Digallic acid (4)	C_14_H_10_O_9_	Ethyl acetate	Yeh et al. (2020)
	3,4-dihydroxybenzoic acid ethy ether (5)	C_9_H_10_O_4_	80% ethanol	Xiang et al. (2010)
	3, 3′-dimethoxy-2, 2′, 4, 4′-tetrahydroxy-diphenylformic acid (6)	N/A	Water	Wei et al. (1999)
	3, 4-dihydroxybenzoic acid (7)	C_7_H_6_O_4_	Chloroform	Yang et al. (2018)
	3,3′-Di-O-methylellagic acid (8)	C_16_H_10_O_8_	acidic ethylacetate	Ito et al. (1990)
	3-((8E,11E)-pentadeca-8,11,14-pentadecatrien-1-yl) phenol (9)	C_21_H_30_O	70% ethanol	Zhang et al. (2019)
	Ellagic acid (10)	C_14_H_6_O_8_	80% acetone	He and Xia. (2007b)
	Ellagic acid-4-O-*α*-L-rhamnpyranoside (11)	C_20_H_16_O_12_	Chloroform	Yang et al. (2018)
	Ellagic acid-4-O-*β*-D-glucopyranoside (12)	N/A	Water	Chang et al. (2017)
	Ellagic acid-4-O-*β*-D-xylopyranoside (13)	N/A	Water	Chang et al. (2017)
	Ethyl gallate (14)	C_9_H_10_O_5_	80% aqueous acetone	He et al. (2008)
	Gallic acid (15)	C_7_H_6_O_5_	80% aqueous acetone	He et al. (2008)
	Geraniin (16)	C_41_H_28_O_27_	Water	Chang et al. (2017)
	4-hydroxybenzaldehyde (17)	C_7_H_6_O_2_	70% ethanol	Zhang (2019)
	4′-hydroxy-3′-methoxy-phenol-*β*-D-[6-O-(4″-hydroxy-3″,5″-dimethoxybenzoate)]glucopyranoside (18)	C_22_H_26_O_12_	70% ethanol	Zhang et al. (2019)
	Isocorilagin (19)	C_27_H_22_O_18_	Water	Chang et al. (2017)
	Methyl brevifolincarboxylate (20)	C_14_H_10_O_8_	ethyl acetate	Chen et al. (2020)
	Methyl gallate (21)	C_8_H_8_O_5_	80% aqueous acetone	He et al. (2008)
	Methyl vanillate (22)	C_9_H_10_O_4_	70% ethanol	Zhang et al. (2019)
	Octyl gallate (23)	C_15_H_22_O_5_	Ethyl acetate	Yeh et al. (2020)
	3-O-galloylquinic acid (24)	N/A	Water	Chang et al. (2017)
	3-O-galloyl quinic acid butyl ester (25)	C_18_H_24_O_10_	80% aqueous acetone	He et al. (2009)
	3-(8-pentadecenyl) phenol (26)	C_21_H_34_O	70% ethanol	Zhang et al., 2019
	Protocatechuic acid (27)	C_7_H_6_O_4_	Ethyl acetate	Kuo et al. (2019)
	Propyl gallate (28)	C_10_H_12_O_5_	Ethyl acetate	Yeh et al. (2020)
	Pyrogallic acid (29)	C_6_H_6_O_3_	80% ethanol	Wu and Xiang (2017)
	Salicylic acid (30)	C_7_H_6_O_3_	80% ethanol	Xiang et al. (2010)
	Sinapic acid (31)	C_11_H_12_O_5_	80% acetone	He and Xia (2007a)
	Urolithin M5 (32)	C_13_H_8_O_7_	Water	Xiao et al. (2022a)
	Vanillic acid (33)	C_8_H_8_O_4_	Ethyl acetate	Yeh et al. (2020)
Flavonoid	Amentoflavone (34)	C_30_H_18_O_10_	80% aqueous acetone	He et al. (2008)
	Astragalin (35)	C_21_H_20_O_11_	Ethyl acetate	Yeh et al. (2020)
	Hyperin (36)	C_21_H_20_O_12_	80% aqueous acetone	He et al. (2008)
	Isoquercitrin (37)	C_21_H_20_O_12_	80% ethanol	Xiang et al. (2010)
	Kaempferol (38)	C_15_H_10_O_6_	80% ethanol	Xiang et al. (2010)
	Kaempferol-3-O-*β*-D-glucopyranoside (39)	N/A	80% aqueous acetone	He et al. (2008)
	Luteolin (40)	C_15_H_10_O_6_	80% ethanol	Xiang et al. (2010)
	Luteolin-7-O-*β*-D-glucoside (41)	C_21_H_20_O_11_	80% ethanol	Xiang et al. (2010)
	Naringin (42)	C_27_H_32_O_14_	Methanol	Wu and Xiang (2017)
	Quercetin (43)	C_15_H_10_O_7_	80% ethanol	Xiang et al., 2010
	Quercetin-3-O-*β*-D-glucoside (44)	C_21_H_19_O_12_	80% ethanol	Xiang et al. (2010)
	Sitoindoside I (45)	C_51_H_90_O_7_	Ethyl acetate	Kuo et al. (2019)
	Tetrahydroamentoflavone (46)	C_30_H_22_O_10_	Ethyl acetate	Kuo et al. (2019)
	3′,4′,7,8-tetrahydroxyflavanone (47)	C_15_H_12_O_6_	80% ethanol	Xiang et al. (2010)
	3,5,7,3′-Tetrahydroxy-4′-methoxyflavanonol (48)	C_16_H_14_O_8_	80% ethanol	Xiang et al. (2010)
	Rutin (49)	C_27_H_30_O_16_	Water/ethanol	Kuo et al. (2015)
Phenylpropanoids	Aesculetin (50)	C_9_H_6_O_4_	Ethyl acetate	Yeh et al. (2020)
	Balanophonin (51)	C_20_H_20_O_6_	70% ethanol	Zhang et al. (2019)
	Cinnamic acid (52)	C_9_H_8_O_2_	70% ethanol	Zhang et al. (2019)
	(+)-(7S,8R,7′S,8′R)-Cinncassin D (53)	C_28_H_28_O_9_Na	70% ethanol	Li et al. (2022)
	(−)-(7R,8S,7′R,8′S)-Cinncassin D (54)	C_28_H_28_O_9_Na	70% ethanol	Li et al. (2022)
	(+)-(7S,8R,7′R,8′S)-Cinncassin D (55)	C_28_H_28_O_9_Na	70% ethanol	Li et al. (2022)
	(−)-(7R,8S,7′S,8′R)-Cinncassin D (56)	C_28_H_28_O_9_Na	70% ethanol	Li et al. (2022)
	Curcasinlignan C (57)	C_18_H_18_O_6_	70% ethanol	Zhang et al. (2019)
	Dihydroconiferyl alcohol (58)	C_10_H_14_O_3_	70% ethanol	Zhang et al. (2019)
	3-ethoxy-1-(4-hydroxy-3-methoxyphenyl)-1-propanone (59)	N/A	70% ethanol	Zhang et al. (2019)
	(7R,8S)-erythro-7,8-bis (4-hydroxy-3-methoxyphenyl)-9-acetyl-7,9-propanediol (60)	C_19_H_22_O_7_	70% ethanol	Zhang et al. (2019)
	(+)-Erythro-(7S,8R)-canariol A (61)	C_29_H_30_O_9_Na	70% ethanol	Li et al. (2022)
	(−)-Erythro-(7R,8S)-canariol A (62)	C_29_H_30_O_9_Na	70% ethanol	Li et al. (2022)
	(+)-erythro-7-O-ethylguaiacylglycerol (63)	C_12_H_18_O_5_	70% ethanol	Zhang et al. (2019)
	(7R,8S)-erythro-guaiacylethoxyglycerol-*β*-O-4′-coniferyl aldehyde ether (64)	C_22_H_26_O_7_	70% ethanol	Chen et al. (2021)
	(7R,8R)-erythro-guaiacylethoxyglycerol-*β*-O-4′-guaiacyl aldehyde ether (65)	C_20_H_24_O_7_	70% ethanol	Zhang (2019)
	(7S,8R)-erythro-guaiacylglycerol-*β*-coniferyl aldehyde ether (66)	C_20_H_22_O_7_	70% ethanol	Zhang (2019)
	(7S,8R)-erythro-3-hydroxyl-4-methoxyl-balanophonin (67)	C_20_H_20_O_6_	70% ethanol	Zhang (2019)
	(7R,8S)-erythro-3-methoxy-4-hydroxy-7- methoxy-8-O-(2′-methoxy-4′-aldehyde-phenyl)-phenylpropanol (68)	C_19_H_22_O_7_	70% ethanol	Zhang (2019)
	(+)-epipinoresinol (69)	C_20_H_22_O_6_	70% ethanol	Zhang (2019)
	Evofolin-B (70)	C_17_H_18_O_6_	70% ethanol	Zhang (2019)
	Ferulic aldehyde (71)	C_10_H_10_O_3_	70% ethanol	Zhang (2019)
	Ficusal (72)	C_18_H_18_O_6_	70% ethanol	Zhang (2019)
	4-hydroxycinnamic acid methyl ester (73)	C_10_H_10_O_3_	70% ethanol	Zhang (2019)
	*β*-hydroxypropiovanilone (74)	N/A	70% ethanol	Zhang (2019)
	Lycocer-nuaside B (75)	N/A	70% ethanol	Zhang (2019)
	(7S)-3-methoxy-4-hydroxy-7-hydromethyl-phenylacetic acid-8-O-(3′-methoxyl-4′-hydroxy) phenyl ester (76)	C_17_H_18_O_7_	70% ethanol	Zhang (2019)
	P-hydroxyphenylferulate (77)	C_16_H_14_O_5_	70% ethanol	Zhang (2019)
	(+)-(7R,8R,7′S,8′R)-Picrasmalignan (78)	C_30_H_30_O_9_Na	70% ethanol	Li et al. (2022)
	(−)-(7S,8S,7′R,8′S)-Picrasmalignan (79)	C_30_H_30_O_9_Na	70% ethanol	Li et al. (2022)
	(+)-(7R,8S,7′S,8′R)-Picrasmalignan (80)	C_30_H_30_O_9_Na	70% ethanol	Li et al. (2022)
	(−)-(7S,8R,7′R,8′S)-Picrasmalignan (81)	C_30_H_30_O_9_Na	70% ethanol	Li et al. (2022)
	(+)-(7S,8R,7′S,8′R)-Picrasmalignan (82)	C_30_H_30_O_9_Na	70% ethanol	Li et al. (2022)
	(−)-(7R,8S,7′R,8′S)-Picrasmalignan (83)	C_30_H_30_O_9_Na	70% ethanol	Li et al. (2022)
	(+)-(7S,8R,7′S,8′S)-Picrasmalignan (84)	C_30_H_30_O_9_Na	70% ethanol	Li et al. (2022)
	(−)-(7R,8S,7′R,8′R)-Picrasmalignan (85)	C_30_H_30_O_9_Na	70% ethanol	Li et al. (2022)
	(+)-pinoresinol (86)	C_20_H_22_O_6_	70% ethanol	Zhang et al. (2019)
	Scoparone (87)	C_11_H_10_O_4_	Water	Wei et al. (1999)
	Scopoletin (88)	C_10_H_8_O_4_	Chloroform	Yang et al. (2018)
	8-O-1 system neolignan (89)	C_19_H_22_O_7_Na	70% ethanol	Zhang et al. (2019)
	(7S,8R)-threo-balanophonin (90)	C_20_H_20_O_6_	70% ethanol	Zhang (2019)
	(+)-Threo-(7R,8R)-canariol A (91)	C_29_H_30_O_9_Na	70% ethanol	Li et al. (2022)
	(−)-Threo-(7S,8S)-canariol A (92)	C_29_H_30_O_9_Na	70% ethanol	Li et al. (2022)
	(+)-Threo-(7R,8R)-canariol B (93)	C_29_H_28_O_9_Na	70% ethanol	Li et al. (2022)
	(−)-Threo-(7S,8S)-canariol B (94)	C_29_H_28_O_9_Na	70% ethanol	Li et al. (2022)
	(7R,8R)-threo-guaiacylethoxyglycerol-*β*-O-4′-coniferyl aldehyde ether (95)	C_22_H_26_O_7_	70% ethanol	Zhang et al. (2019)
	(7R,8S)-threo-guaiacylethoxyglycerol-*β*-O-4′-guaiacyl aldehyde ether (96)	C_20_H_24_O_7_	70% ethanol	Chen et al. (2021)
	(7S,8R)-threo-1'-[3′-hydroxy-7-(4-hydroxy-3-methoxyphenyl)-8-hydro-xymethyl-7,8 dihydrobenzofuran] acrylaldehyde (97)	C_19_H_18_O_6_	70% ethanol	Zhang et al. (2019)
	Threo-1-(4-hydroxy-3-methoxyphenyl)-1-methoxy-2-{4-[1-formyl-(E)-vinyl]-2-methoxyphenoxy}-3-propanol (98)	C_21_H_24_O_7_	70% ethanol	Zhang et al. (2019)
	(7S,8R)-threo-lycocernuaside B (99)	C_19_H_20_O_6_	70% ethanol	Zhang (2019)
	(7R,8S)-threo-3-methoxyl-4-hydroxyl-balanophonin (100)	C_20_H_20_O_6_	70% ethanol	Zhang (2019)
	(7S,8S)-threo-3-methoxy-4-hydroxy-7- methoxy- 8-O-(2′-methoxy-4′-aldehyde-phenyl)-phenylpropanol (101)	C_19_H_22_O_7_	70% ethanol	Chen et al. (2021)
	Vladinol-D (102)	C_20_H_22_O_7_	70% ethanol	Zhang et al. (2019)
Triterpenoid	*α*-Amyrin (103)	C_30_H_50_O	Ethanol	Tamai et al. (1989)
	*β*-amyrin (104)	C_30_H_50_O	Ethanol	Tamai et al. (1989)
	3-epi-α-amyrin (105)	C_30_H_50_O	Ethanol	Tamai et al. (1989)
	3-epi-*β*-amyrin (106)	C_30_H_50_O	Ethanol	Tamai et al. (1989)
	Olean-12-ene-3*α*,16*β*-diol (107)	—	Ethanol	Tamai et al. (1989)
	Urs-12-ene-3*β*,16*α*-diol (108)	C_30_H_50_O_2_	Ethanol	Tamai et al. (1989)
	Urs-12-ene-3*α*,16*β*-diol (109)	C_30_H_50_O_2_	Ethanol	Tamai et al. (1989)
Other types	CPS1 (110)	—	Water	Zeng et al. (2015)
	CPS2 (111)	—	Water	Zeng et al. (2015)
	CPS3 (112)	—	Water	Zeng et al. (2015)
	Myo-inositol (113)	C_6_H_12_O_6_	Chloroform	Yang et al. (2018)
	(1′R, 3′S, 5′R, 8′R, 2Z, 4E)-dihydrophaseic acid-3′-O-*β*-D-glucopyranoside (114)	C_21_H_32_O_10_	Chloroform	Yang et al. (2018)
	(1′S, 3′R, 5′S, 8′R, 2Z, 4E)-dihydrophaseic acid-3′-O-*β*-D-glucopyranoside (115)	C_21_H_32_O_10_	Chloroform	Yang et al. (2018)

### 3.1 Phenolic acid

**FIGURE 2:**
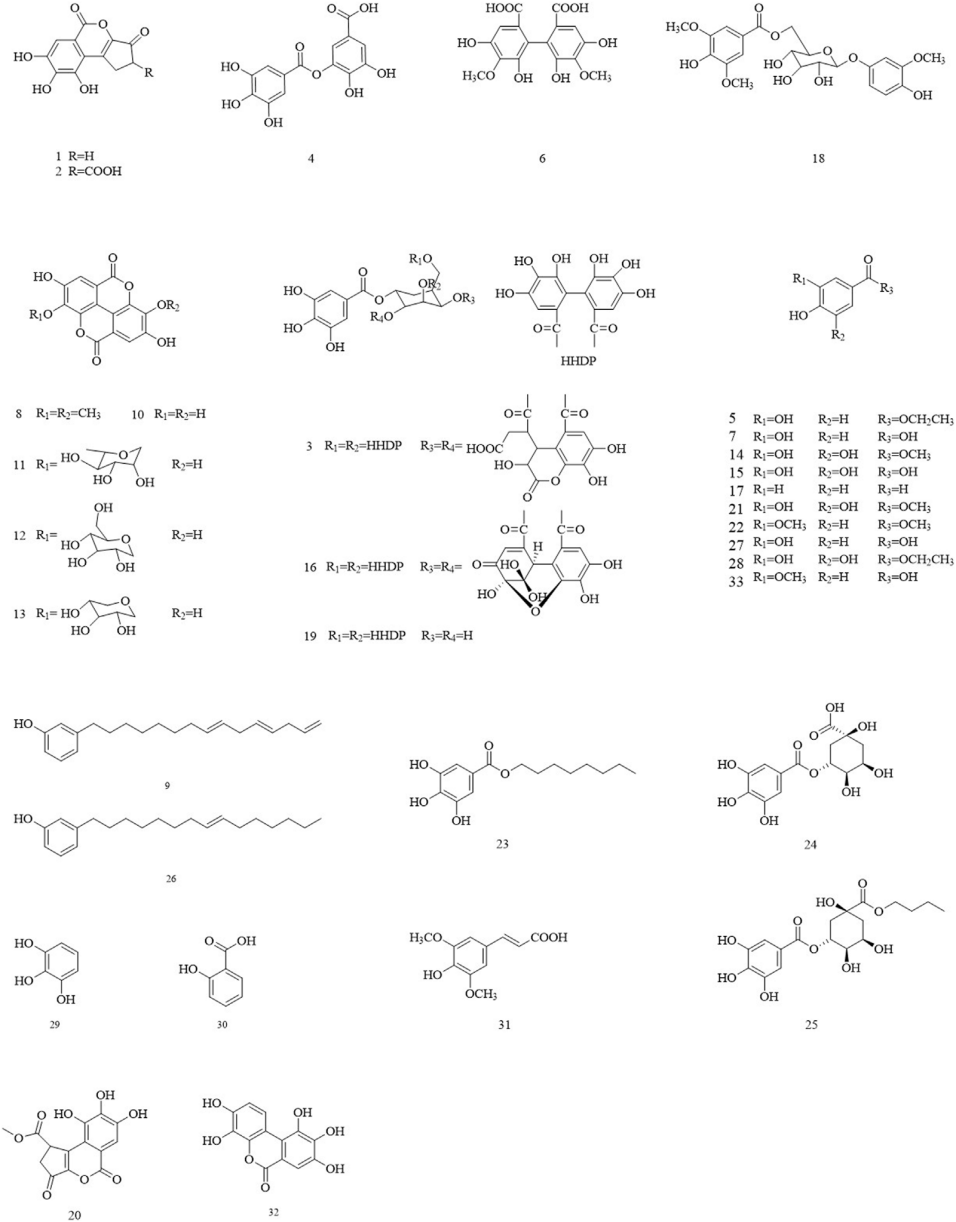
The structures of the polyphenol in Chinese olive.

Phenolic acids are the most abundant phenolic compounds in Chinese olives. They include gallic acid, ellagic acid, salicylic acid, vanillic acid, sinapic acid, isocorilagin, among others. Their specific structure is illustrated in Figure 2. Studies have shown that gallic acid (166 mg/100 g FW) and ellagic acid (87.8 mg/100 g FW) were the main phenolic compounds in Chinese olives, accounting for 32.9% and 17.4% of the total phenolics content, respectively.

### 3.2 Flavonoid

**FIGURE 3:**
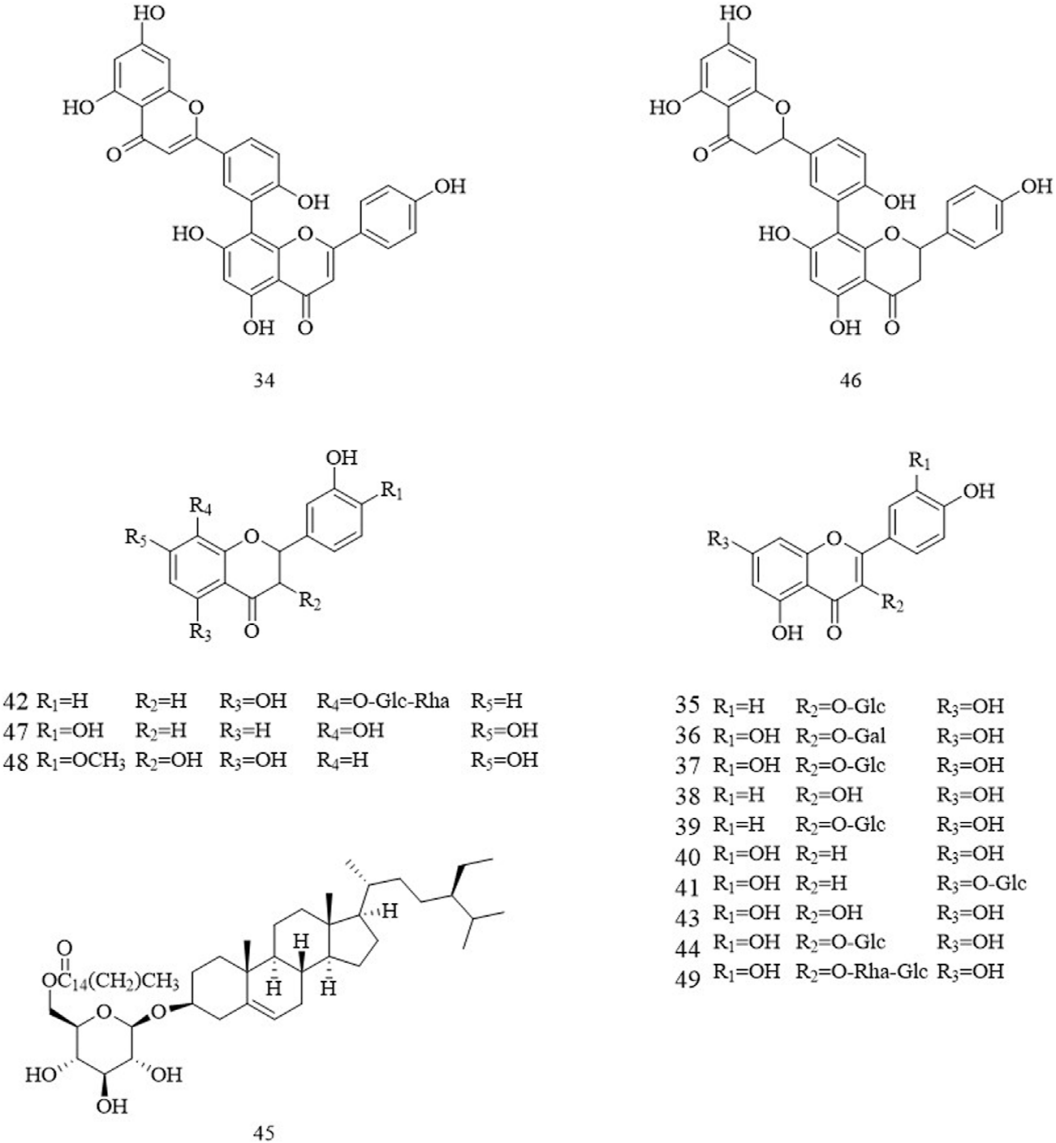
The structures of the flavone in Chinese olive.

Flavonoids are one of the main plant metabolites in Chinese olives. They mainly exist in fruits, roots, stems, leaves, and other parts. Flavonoids in Chinese olives include flavonols, dihydro flavonoids, isoflavones, chalcones, and their glycosides, etc. Currently, the flavonoids that have been isolated from Chinese olives are brevifolin, hyperin, isoquercetin, astragalin, luteolin-7-O-glucoside, quercetin, kaempferol, sitoindoside I, tetrahydroamentoflavone, amentoflavone and kaempferol-3-glucopyranoside (Figure 3).

### 3.3 Phenylpropanoid

**FIGURE 4:**
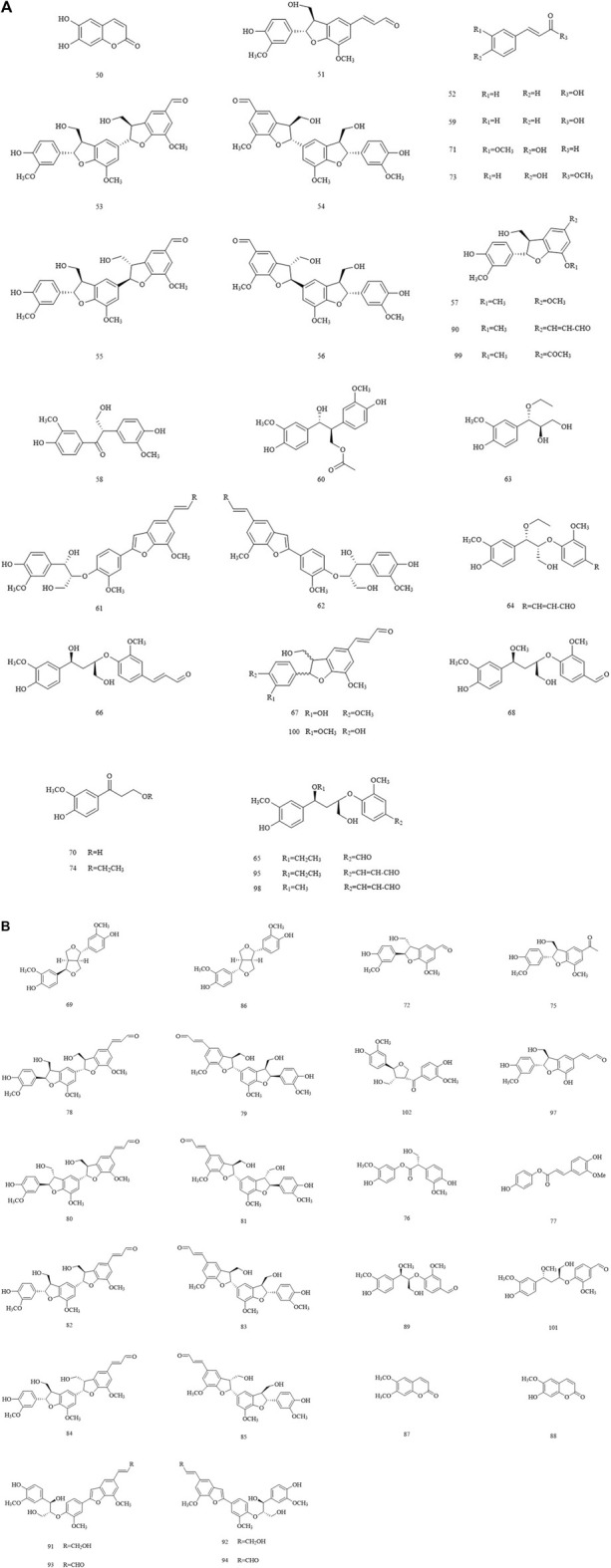
**(A)** The structures of the phenylpropanoids in Chinese olive. **(B)** The structures of the phenylpropanoids in Chinese olive.

Phenylpropanoids, including simple phenylpropanoids, lignans, and coumarins, have been isolated and characterized from Chinese olives (Figure 4). Among them, the isolated coumarins include scopoletin, aesculetin, and scoparone. Several structurally novel neolignins were consistently isolated from Chinese olives in recent years. The latest studies have revealed that nine pairs of new neolignin enantiomers, which belong to the benzofuran type, were identified and isolated from Chinese olives.

### 3.4 Triterpenoid

**FIGURE 5:**
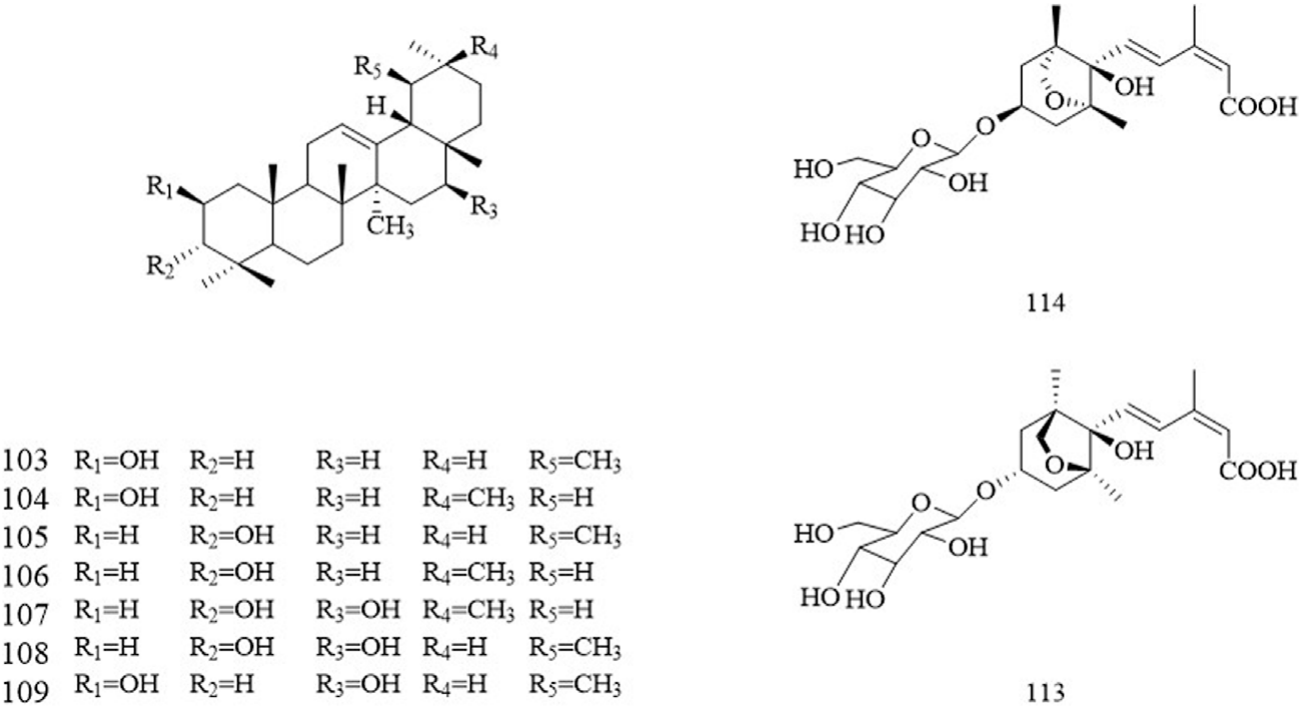
The structures of the terpenoids and other compounds in Chinese olive.

The terpenoids in Chinese olive are mainly triterpenes, which are a type of compound with 30 carbon atoms in the mother nucleus. Current triterpenoids isolated from Chinese olive are 3-epi-*α*-amyrin, Urs-12-ene-3*α*, 16*β*-diol, Urs-12-ene-3*β*, 16α-diol, *β*-amyrin, 3-epi-*β*-amyrin, Olean-12-ene-3*α*, 16*β*-diol (Figure 5).

### 3.5 Other bioactive compound

Phenols are considered one of the phytochemical groups providing the beneficial properties of Chinese olives. Furthermore, three polysaccharides, CPS1, CPS2, and CPS3, from Chinese olive has been recently isolated. CPS1 and CPS3 are neutral polysaccharides with *α*- and *β*-glycosidic linkages and CPS2 is a pectic polysaccharide mainly linked by *β*-glycosidic linkages. In addition, monoterpenoids and sesquiterpenoids volatile components have been isolated in Chinese olives, such as two sesquiterpenes (1′S, 3′R, 5′S, 8′R, 2Z, 4E)-dihydrophaseic acid-3′-O-*β*-d-glucopyranoside, (1′R, 3′S, 5′R, 8′R, 2Z, 4E)-dihydrophaseic acid-3′-O-*β*-d-glucopyranoside. Due to Chinese olive chemical structure diversity, variation in bioactive compounds leads to their different properties and biological activities, which has attracted increasing research interest.

### 3.6 Biosynthesis

**FIGURE 6:**
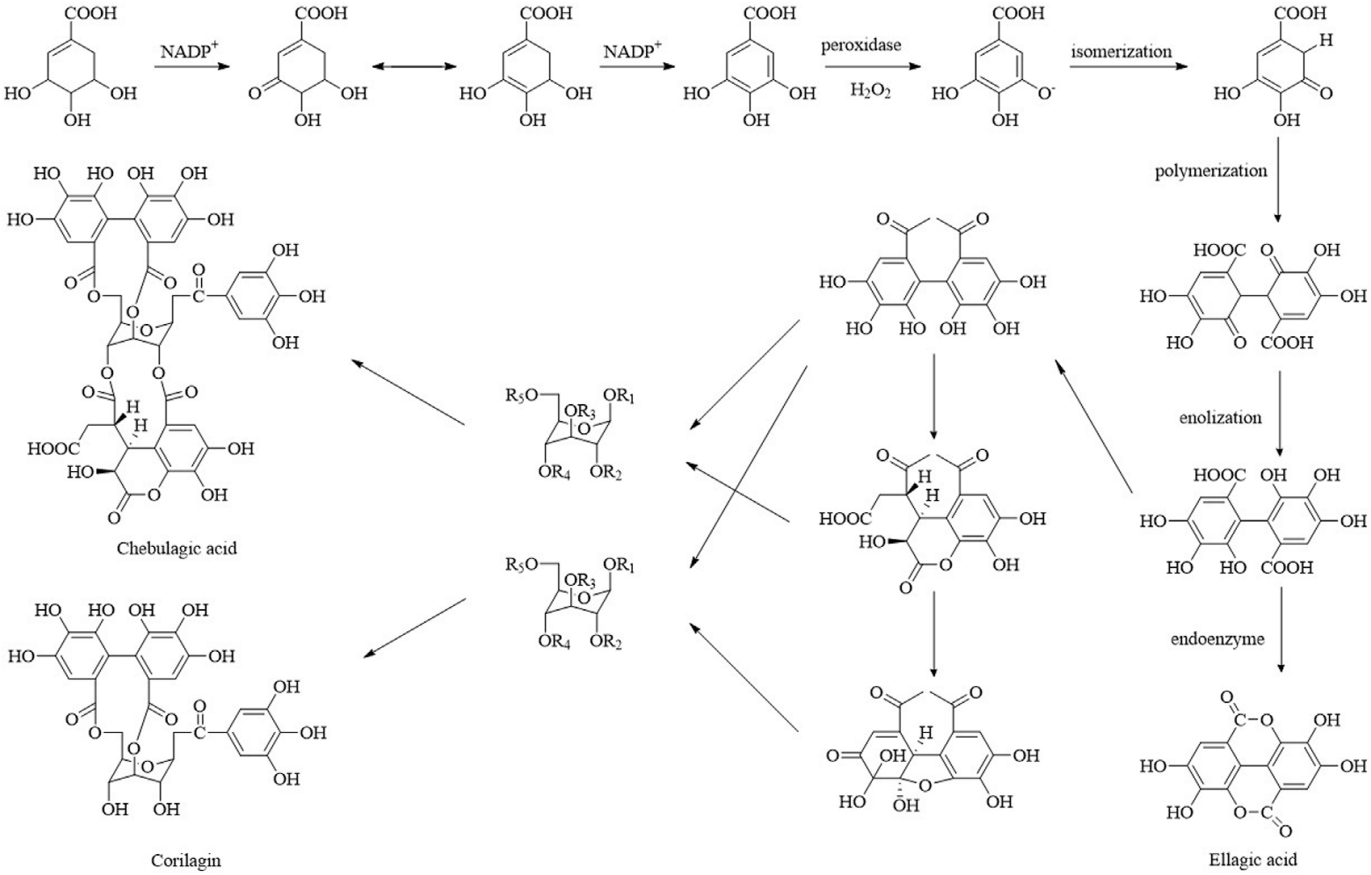
Biosynthetic pathways of ellagic acid, corilagin, and chebulagic acid.

Natural products are mainly rich in plant secondary metabolites (Sohn et al., 2022). Secondary metabolites are the result of plant adaptation to the ecological environment in the long-term evolution process (Mohaddab et al., 2022). Plant secondary metabolites are characterized by their diversity and abundance, their diverse structures, and significant biological activities. Phenolic compounds comprise the largest group of known secondary metabolites in plants (Dias et al., 202a). Fruits are rich in phenols, and thus are one of the main dietary sources for human phenol intake. Chinese olive, as a fruit, contains a high concentration of phenolic compounds. Phenolic compounds are mainly synthesized through the shikimic acid pathway, the phenylpropanoid pathway, and the flavonoid pathway, and their precursors are derived from intermediate sugar metabolites (Figure 6) (Wu et al., 2022). In the biosynthetic pathway of phenolics, the shikimate pathway is an important bridge connecting the sugar metabolism with the downstream secondary metabolism, while the phenylpropanoid and flavonoid pathways are the primary links (Maeda and Dudareva, 2012). Shikimic acid undergoes transamination to phenylalanine to produce intermediate products such as cinnamic acid, coumaric, and ferulic acid through a series of related reactions. These are further converted to coumarins, lignans, lignin, flavonoids, condensed tannins, and other secondary metabolites (De Araújo et al., 2021). Flavonoids are a large phenolic compound class carrying a phenyl chromone ring. They are synthesized through the phenylpropanoid pathway. Flavonoid biosynthesis occurs at the junction of the shikimate pathway and acetate pathway, with the shikimate pathway providing the coumarin CoA synthesis substrate and the acetate pathway themalonyl—CoA moieties for the C2 elongation reaction catalyzed by chalcone synthase (Dias et al., 2021b). Chinese olives contain many flavonoids, among which hyperoside is a product of the flavonol branch in the phenylpropanoid pathway. Quercetin is a direct precursor of hyperoside, and flavonoid 3-O-galactosyltransferase (UF3GaT) is responsible for hyperoside final production by catalyzing the galactosyl group of quercetin on the O group at position 3 (Huang, 2017). These flavonoids exhibit significant human health benefits and are promising additives in modern food, nutrition, and medicinal products. The green, unripe fruit phenolic compounds include mainly gallic acid, ellagic acid, chebulagic acid, and corilagin, among others. Studies have shown that the shikimate pathway is responsible for the production of gallic acid. Shikimic acid produces 3-dehydroshikimic acid catalyzed by shikimate dehydrogenase. Subsequently, 3-dehydroshikimic acid undergoes enolation, catalyzed by 3-dehydroshikimic acid dehydrogenase, to produce gallic acid. Gallic acid can be esterified to form ellagic acid in the presence of peroxidase, H_2_O_2_. Under the same conditions, gallic acid and its polymer combined with glucose esters could form corilagin and chebulagic acid (Sharifi-Rad et al., 2022). In addition, in the latest studies, nine pairs of new enantiomers of benzofuran neolignans were identified in Chinese olives (Li et al., 2022). Lignans, a very important natural secondary metabolite, have been found in various plants as mixtures of enantiomers. Research reports demonstrate that if two isozymes with opposite stereospecificity simultaneously exist in plants, a pair of enantiomers may be formed and be present. Whether this is the case for Chinese olive remains to be further verified and determined. Many studies on phytochemical biosynthetic pathways have revealed that phenolic compounds synthesis and distribution are significantly different in cultivars, affected by cultivation conditions, etc. At the same time, phenolic compounds play an important role in fruit flavor quality, usually affecting fruit flavor in terms of color and flavor. By comparing the phenolic content changes in three different Chinese olive varieties during ripening, significant differences were observed in the phenolic content, which also caused the differences in flavor between the varieties. The varieties with crisp fruit and a strong taste had higher total phenol and flavonoid content. In contrast, the varieties with sweet fruit and no astringency had the lowest total phenols and flavonoid content. The varieties with rough flesh and light taste had a significantly higher total lignin content compared to the varieties with a crisp and strong taste (2.34—fold higher) and the varieties with sweet and non-astringent fruit (4.18—fold higher). Among them, the difference in ellagic acid, hyperoside, ferulic acid, and sinapic acid contents may be an important factor contributing to the fruit flavor differences (Lai et al., 2022). In addition, lighting conditions and stress are major factors affecting plants’ phenolic content, directly affecting phenolic compounds metabolism in plants. By knowing the biosynthetic pathway of the various compounds, their precursors, and the various intermediates, we can artificially inject precursors or intermediates into plants to increase the accumulation and yield of compounds of interest to achieve artificial compound synthesis control and directional cultivation (Singh et al., 2022). These findings are of great significance to the improvement of fruit flavor and resource utilization and can also provide a reference for high-quality cultivation and breeding innovation of the Chinese olive.

## 4 Health benefits

**FIGURE 7:**
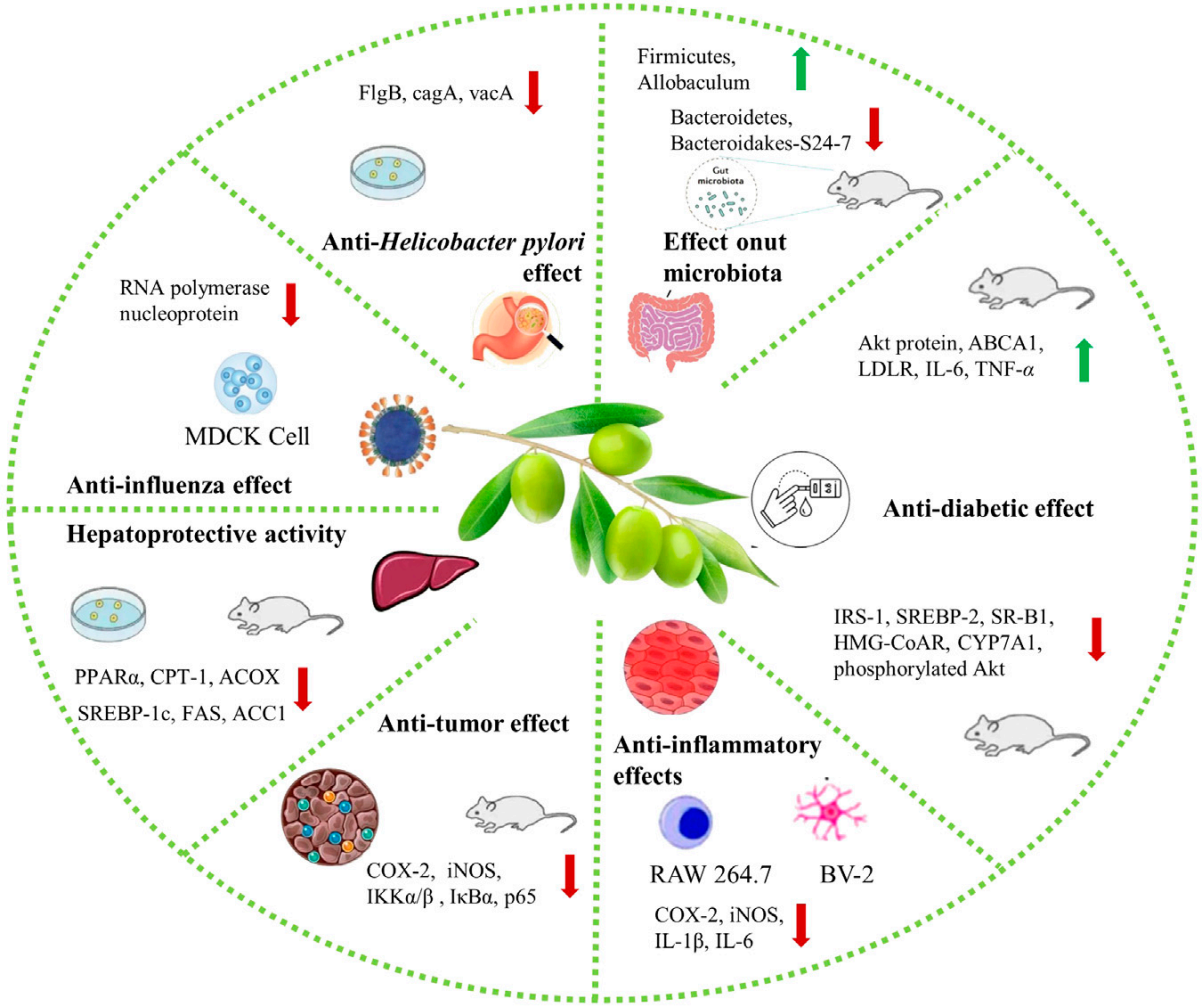
Health benefits of Chinese olive (The red arrow indicates upward adjustment; the green arrow indicates downward adjustment).

With the link between diet and health strengthening, several food products have emerged as possessing potential health benefits. The Chinese olive also exhibits health benefits as an “affinal drug and diet” plant. The current scientific research has profiled several of its bioactive components and properties and confirmed certain health benefits features (Figure 7). The Chinese olive health-promoting properties and mechanism of action determined in cell and animal studies are summarized in Table 4.

**TABLE 4:** Exhibition of health benefits of different components of Chinese olive on various diseases.

Pharmacological activities	Compounds/extracts	*In vivo* or *in vitro*	Model/method	Result/mechanism	References
Anti-*Helicobacter pylori* effect	Water extract and ethyl acetate extract of Chinese olive	*in vitro*	Standard strains and clinically resistant strains	Downregulation of virulence genes, such as urelI, alpA, alpB, flgA, cagA, vacA	Yan et al. (2022)
Anti-influenza virus A	Scopoletin and isocorilagin	*in vitro*	MDCK cells	Scopoletin and isocorilagin displayed significant anti-influenza virus A activities with IC_50_ = 22.9 ± 3.7 and IC_50_ = 5.42 ± 0.97 μg/mL, respectively	Yang et al. (2018)
Anti-influenza virus A	Isocorilagin	*in vitro*	MDCK cells	Mechanistic studies revealed that Chinese olive inhibited neuraminidase activity of IAV and directly influenced the virus release	Chen et al. (2020)
Anti-influenza virus A	Brevifolincarboxylate	*in vitro*	MDCK cell	Brevifolincarboxylate inhibited the replication of influenza A virus by targeting PB2 cap-binding domain	Chen et al., 2020
Anti-influenza virus A	Ethyl acetate extract	*in vitro*	N/A	The ethyl acetate extract of Chinese olive has strongly inhibited the HIV-1 glycoprotein subunit 41 six-helix bundle formation	Duan et al. (2013)
Hepatoprotective activities	Ethyl acetate fraction of Chinese olive	*in vivo* and *in vitro*	FL83B mouse hepatocytes C57BL/6 mice fed a 60% high-fat diet	CO-EtOAc suppressed the mRNA levels of fatty acid transporter genes (CD36 and FABP) and lipogenesis genes (SREBP-1c, FAS, and ACC1), but upregulated genes that govern lipolysis (HSL) and lipid oxidation (PPARα, CPT-1, and ACOX)	Yeh et al. (2018)
Hepatoprotective activities	Brevifolin, ellagic acid and 3,3′-di-O-methylellagic acid	*in vivo*	Carbon tetrachloride -induced cytotoxicity in primary cultured rat hepatocytes	Brevifolin, ellagic acid and 3,3′-di-O-methylellagic acid from Chinese olive have been shown to reduce liver damage in mice caused by carbon tetrachloride	Ito et al. (1990)
Anti-diabetic effect	Ethyl acetate fraction of fruit extract	*in vivo*	mice fed a high-fat diet	Chinese olive fruit regulates glucose utilization by activating AMP-activated protein kinase	Yeh et al. (2017)
Anti-diabetic effect	Ethyl acetate fraction of fruit extract	*in vivo*	mice fed a high-fat diet	Chinese olive fruit may ameliorate metabolic dysfunction in diabetic rats under HFD challenge	Yeh et al. (2020)
Anti-diabetic effect	Chinese olive extract	*in vitro*	A bovine serum albumin (BSA)-glucose glycosylation reaction system	The results showed that the Chinese olive extract showed good inhibitory effect on AGEs	Kuo et al. (2015)
Anti-inflammatory effects	Balano-phonin, (7S,8R)-threo-1′-[3′-hydroxy-7-(4-hydroxy-3-methoxyphenyl)-8-hydro-xymethyl-7,8 dihydrobenzofuran] acrylaldehyde, erythro-guaiacylethoxy glycerol-β-O-4′-guaiacyl aldehyde ether and ferulic aldehyde	*in vitro*	LPS-induced microglial BV-2 cells	Balano-phonin, (7S,8R)-threo-1′-[3′-hydroxy-7-(4-hydroxy-3-methoxyphenyl)-8-hydro-xymethyl-7,8 dihydrobenzofuran] acrylaldehyde, erythro-guaiacylethoxy glycerol-β-O-4′-guaiacyl aldehyde ether and ferulic aldehyde in Chinese olive could dose-dependently reduce the expression levels of pro-inflammatory mediator iNOS and COX-2 expressions induced by LPS in BV-2 cells	Zhang et al. (2019)
Anti-inflammatory effects	Benzofuran Neolignans	*in vitro*	RAW 264.7 macrophages cells	(+)-(7R,8R,7′S,8′R)-Picrasmalignan and (−)-(7S,8S,7′R,8′S)-Picrasmalignan could block the nuclear translocation of NF-κB and reduce the expression of pro-inflammatory mediators COX-2, iNOS, IL-1β, and IL-6 to exert anti-inflammatory effects	Li et al. (2022)
Anti-inflammatory effects	Ethyl acetate fraction of Chinese olive	*in vitro*	RAW 264.7 macrophages cells	Ethyl acetate fraction of Chinese olive showed that the active compounds with anti-inflammatory effect were sitoindoside I, amentoflavone, tetrahydroamentoflavone, and protocatechuic acid	Kuo et al. (2019)
Anti-tumor effect	Methanol-ethyl acetate partitioned fraction from Chinese olive fruits	*in vivo*	0.2 mL of CT26 cell suspension was subcutaneously injected into the right hind legs of the mice	The methanol-ethyl acetate partitioned fraction from Chinese olive fruits inhibits cancer cell proliferation and tumor growth by promoting apoptosis through the suppression of the NF-κB signaling pathway	Hsieh et al. (2016)
Regulation of gut microbiota associated diseases	Chinese olive extract	*in vivo*	mice fed a high-fat diet	The showed significant increases of *Firmicutes* and *Verrucomicrobia*, but a decrease of *Bacteroidete*s in all Chinese olive-fed mice. Chinese olive gavage in a low dose or a medium dose caused a significant increase in the proportion of *Akkermansia*	Zhang et al. (2018)

### 4.1 Anti-*Helicobacter pylori* effect

*Helicobacter pylori* (*H. pylori*) is an infectious gram-negative microaerobic bacterium. It is associated with various digestive diseases, such as gastric cancer, gastric ulcers, duodenal ulcer, and gastritis (Malfertheiner et al., 2023). In the latest United States Department of Health and Human Services (HHS) carcinogen report, *H. pylori* is listed as a carcinogen. At present, the resistance and adverse reactions towards *H. pylori*-related drugs, such as clarithromycin, metronidazole, and levofloxacin, are increasing (Bubić et al., 2023). Therefore, finding safe, reliable, and effective drugs to treat the disease is particularly important.

Chinese olive exhibits promising biological activity against *H. pylori*. The Chinese olive inhibitory effect on *H. pylori*, including the effect of chemical composition, *in vitro* antibacterial activity, and a preliminary mechanism of antibacterial action of Chinese olive extracts from different regions and using different solvents, have been investigated. Among them, the water and ethyl acetate extracts had certain inhibitory and bactericidal effects on *H. pylori* standard and clinically resistant strains. The broth microdilution method was used to measure the minimum inhibitory concentration (MIC) and minimum bactericidal concentration (MBC) of the two extracts. The MIC of water extract and ethyl acetate extract was 156–625 μg/mg, and MBC was 78–1,250 μg/mg. The *H. pylori*’s shape can change from helical to spherical due to changes in its living environment, including insufficient nutrient supply, dryness, lack of antioxidant protection, or after antimicrobial treatment. Scanning electron microscopy (SEM) and transmission electron microscopy (TEM) showed that both Chinese olive extracts induced morphological changes in *H. pylori*. Specifically, its shape changed from helical to spherical. Separating the cell membrane from the cell wall was observed as well as producing intracellular vesicles and leakage of cell contents to form vacuolar cells. Furthermore, urease has been shown to be a virulence factor for *H. pylori* and is essential for its survival in a strongly acidic environment. The effect of two Chinese olive extracts on urease was measured by the Berthelot method with acetohydroxamic acid as a positive control. The Chinese olive extracts could inhibit the *H. pylori* urease in a dose-dependent manner. The IC_50_ value of the positive control group, water extract, and acetic acid extract were 40.9, 1,093, and 332.90 μg/mL, respectively. Furthermore, Chinese olive aqueous extracts can downregulate the flagellum synthesis gene (*flgB*) expression, while ethyl acetate extract can downregulate the expression of cytotoxin-associated gene A (*cagA*) and vacuolating cytotoxin gene (*vacA*) (Yan et al., 2022). In summary, Chinese olive can reduce the risk of gastric diseases by inhibiting *H. pylori* and bring health benefits with its consumption.

### 4.2 Anti-influenza effect

**FIGURE 8:**
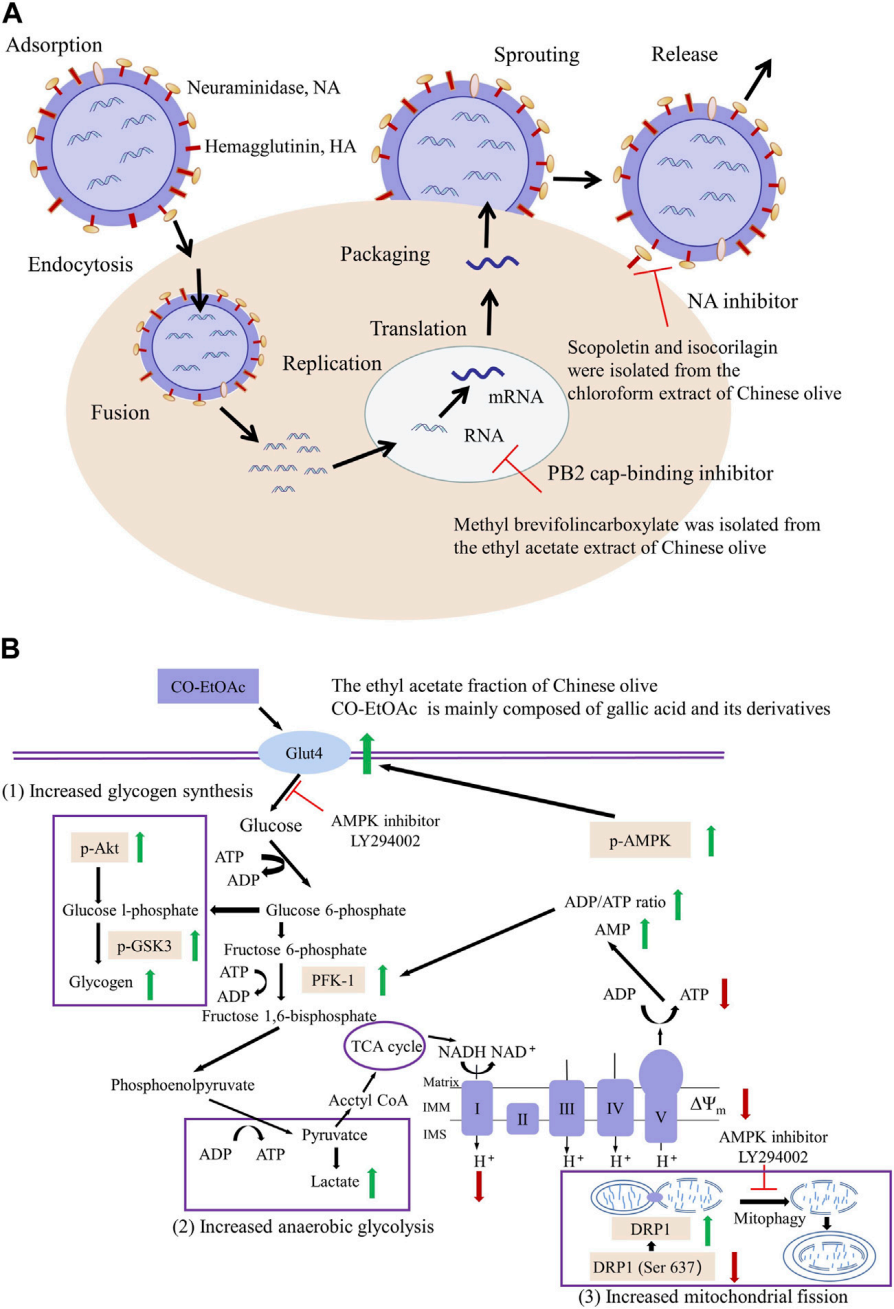
Potential mechanism of Chinese olive against influenza and diabetes. **(A)** The potential mechanism of anti-influenza effect of Chinese olive. **(B)** The potential mechanism of anti-diabetic effect of Chinese olive.

Influenza is a contagious respiratory disease caused by the influenza virus and is a public health problem of global concern. In the context of COVID-19 pandemic, influenza prevention and treatment are becoming increasingly important due to its similar symptoms to COVID-19 (Da Costa et al., 2020; Bonacina et al., 2023). Twelve compounds were isolated from Chinese olive chloroform extract using macroporous adsorption resin, silica gel, Sephadex LH-20, Toyopearl HW-40F, and reversed-phase C18 columns. The isolated compounds’ anti-influenza activity was determined by the 3-(4,5-dimethylthiazol-2-yl)-2, 5-diphenyltetrazolium bromide (MTT) colorimetric assay, with ribavirin as the control. Among them, isocorilagin and scopoletin exhibited inhibitory activity against influenza virus A/Puterto Rico/8/34 H274Y (H1N1) with IC_50_ values of 5.42 ± 0.97 μg/mL and 22.9 ± 3.7 μg/mL, respectively (Yang et al., 2018). Subsequently, isocorilagin inhibitory activity and mechanism of action were further investigated. Isocorilagin demonstrated excellent antiviral activity on different influenza A virus (IAV) strains with no apparent cytotoxicity. The cells cytopathic effect (CPE) phenomenon results confirmed that isocorilagin could prevent damage induced by IAV infection in Madin-Darby canine kidney (MDCK) cells. The mechanism of isocorilagin inhibiting influenza was further analyzed *in vitro*. It strongly affected the release of progeny virions rather than influencing virus entry or replication. Finally, the isocorilagin inhibitory effect on neuraminidase (NA) was verified by molecular docking. In summary, isocorilagin can strongly bind to the NA active site through hydrogen bonds and van der Waals forces in the amino acid residues (Glu119, Asp151, Arg118, Arg156, Arg224, Glu276, and Asn294) to block the virion progeny virion release to exert its anti-influenza effect (Chen et al., 2020). Furthermore, methyl brevifolincarboxylate (MBC), extracted from Chinese olives, similarly exhibits anti-influenza properties. The effect of MBC against the influenza virus was systematically evaluated *in vitro*. MBC exhibited inhibitory effects on different strains of IAV, including A/Puerto Rico/8/34 (H1N1) (PR8) (IC_50_ = 27.16 ± 1.39 μM) and H3N2 (IC_50_ = 33.41 ± 2.34 μM). Additionally, MBC demonstrated a good inhibitory effect on influenza virus hemagglutinin (HA)-mRNA and nucleoprotein (NP) expression levels in PR8 cells in a dose-dependent manner. This indicated that MBC could effectively inhibit influenza virus replication in infected cells. However, no obvious effect on the entry and release of the virus was observed. The MBC mechanism of action was preliminarily investigated by indirect immunofluorescence assay, mini-replicon assay, homogeneous time-resolved fluorescence (HTRF) assay, and molecular docking approaches. According to those, MBC exhibited a strong binding capacity to PB2 cap protein, forming a hydrogen bond to inhibit RNA polymerase activity, which could explain its antiviral activity (Chen et al., 2020). The above results suggest that Chinese olive may be effective against influenza strains not being susceptible to virus-induced resistance. This provides a broad application prospect for the use of Chinese olives to manage the influenza virus (Figure 8).

Except for the influenza virus, Chinese olive was shown to have an inhibitory effect on human immunodeficiency virus (HIV). The formation of the HIV-1 glycoprotein subunit 41 six-helix bundle is critical for viral fusion with the target cell. It prevents the virus from entering target cells if it cannot be formed, thereby protecting immune cells from infection. The ethyl acetate extract of Chinese olive strongly inhibits the HIV-1 glycoprotein subunit 41 six-helix bundle formation. Four fractions of Chinese olive ethyl acetate extract, determined by high-speed counter-current chromatography, displayed significant inhibitory effects on HIV-1 IIIB infection. One of the compounds was identified as 5-hydroxymethylfurfural (Duan et al., 2013). Based on Chinese olive properties against influenza and HIV virulence, it can be widely used in daily healthcare to reduce disease burden and improve health and quality of life.

### 4.3 Hepatoprotective effect

Non-alcoholic fatty liver disease (NAFLD) is the most common chronic liver disease worldwide. There is a lack of effective, safe, and affordable drugs to treat NAFLD. Compared with the use of conventional medicine, more attention has been shifted towards the treatment of NAFLD due to its multi-target action mechanism. The potential mechanism and therapeutic effect of Chinese olive ethyl acetate fraction (CO-EtOAc) on NAFLD induced by a high-fat diet (HFD) were investigated (Yeh et al., 2018). Using FL83B mouse hepatocytes as an *in vitro* model, the addition of 600 μM oleic acid (OA) for 24 h resulted in lipid accumulation in the cells, while treated with 50–400 μg/mL Treatment with Chinese olive extract for 21 h, significantly decreased lipid accumulation in a concentration-dependent manner. Thus, Chinese olive extract could reduce cell lipid accumulation induced by OA. Mechanistic studies indicated that the fatty acid transporter genes (FABP and CD36) and lipogenesis genes (SREBP-1c, FAS, and ACC1) mRNA levels were suppressed while genes regulating lipolysis (HSL) and lipid oxidation (PPARα, CPT-1, and ACOX) were upregulated. AMPK-mediated phosphorylation of target substrates plays a crucial role in regulating and maintaining cellular homeostasis. Chinese olive extract treatment increased the protein expression of phosphorylated AMPK, CPT-1, ACC1, and PPARα but resulted in the downregulation of mature SREBP-1c and FAS. Subsequently, the regulatory effect of Chinese olive on liver lipid metabolism was further investigated using an animal model, C57BL/6 mice fed with 60% HFD. HFD-fed mice exhibited a significant increase in body and epididymal adipose tissue weight, liver total cholesterol and triglyceride levels, and blood glucose, insulin, and triglycerides. These changes were attenuated after treatment with Chinese olive extract, and the HFD-induced changes in hepatic appearance and increase in abdominal fat accumulation were ameliorated. In summary, the Chinese olive extract can regulate lipid accumulation *in vitro* and *in vivo*. It can protect the liver from HFD-induced hepatic steatosis by reducing lipid gene expression through an AMPK-mediated mechanism. Additionally, Chinese olive compounds such as brevifolin, ellagic acid, and 3,3′-di-O-methylellagic acid have been shown to reduce liver damage in mice caused by carbon tetrachloride (Ito et al., 1990).

### 4.4 Anti-diabetic effect

Diabetes is a chronic multifactorial metabolic disease. Based on previous research, the Chinese olive extract has promising effects in treating or improving diabetes. Administration of 150 mg/kg Chinese olive extract to a rat fed with HFD combined with a streptozotocin (STZ) injection demonstrated that Chinese olive extract could significantly reduce body weight, epididymal adipose tissue, and blood glucose levels. It additionally increased antioxidant enzyme (SOD, GPx, CAT) activity and decreased lipid peroxidation, resulting in a reversal of the hepatic oxidative damage induced by HFD feeding. Further evaluation of the Chinese olive extract’s effects on the insulin signaling pathway pointed to a decrease in the phosphorylated IRS-1 and the upregulation of the phosphorylated Akt protein in the liver. Moreover, diabetes is often accompanied by a widespread dysregulation of lipid metabolism and cholesterol homeostasis. The hepatic cholesterol level of diabetic control group (DC) rats was significantly increased, while it was significantly decreased after treatment with 150 mg/kg Chinese olive extract. Investigating the expression of genes involved in cholesterol transport, biosynthesis, and degradation, Chinese olive extract suppressed SREBP-2, SR-B1, HMG-CoAR, and CYP7A1 mRNA levels but also increased the expression of genes such as ABCA1 and LDLR, that control cholesterol efflux and uptake. Diabetes, based on numerous studies, is a disease with hyperglycemia as the primary manifestation and an inflammatory disorder. The mRNA and protein levels of IL-6 and TNF-*α* in the DC group were significantly increased compared with the control group but decreased after treatment with Chinese olive extract (Yeh et al., 2017). Diabetes is also closely linked with the accumulation of AGEs. Non-enzymatic protein glycosylation is the formation of stable non-enzymatic glycosylation end products (AGEs) through a series of reactions between free amino groups in macromolecules such as proteins and aldehyde groups in reducing sugars. The anti-glycosylation activity of different Chinese olive solvent extracts was determined by using bovine serum albumin (BSA)-glucose glycosylation reaction system, using aminoguanidine as the positive control. Chinese olive extract demonstrated a good inhibitory effect on AGEs, with extracts obtained with water/ethanol solvent exhibiting the strongest inhibitory effects. In contrast, acetone extracts and ethyl acetate extracts exhibited the weakest inhibitory effect. Thus, Chinese olives extracted with water/ethanol significantly inhibited protein glycosylation, which may be related to their phenolic composition (Kuo et al., 2015).

Insulin resistance is a hallmark of type 2 diabetes and is currently considered an initiating factor in type 2 diabetes onset. Chinese olive extract improved insulin resistance in rats, possibly by affecting AMPK activation and the promotion of mitochondrial fission. Four-week-old C57BL/mice fed with HFD were used to establish an experimental model. The Chinese olive extract’s effects on blood glucose were evaluated by measuring the body weight, blood biochemical parameters, and the hepatic inflammatory response. Chinese olive extract reduced body weight, improved FBG fast blood glucose levels, and reduced hepatic lipid accumulation induced by HFD in C57BL/mice. An L6 myotube model was employed to define further the molecular mechanism underlying the Chinese olive extract-mediated blood glucose reduction in HFD-fed mice. The highest activity of the Chinese olive extract was observed at a concentration of 400 μg/mL. It promoted glucose uptake in myotubes, lasting 0.5–0.6 h. At the same time, an increase in glucose transporter 4 (GLUT4) translocation can also be observed. Additionally, the AMPK phosphorylation level was decreased, and phosphorylated Akt (Ser-473) and phosphorylated Akt (Thr-308) were increased. In brief, Chinese olive extract was shown to improve insulin resistance in rats, possibly through effects on AMPK activation and promotion of mitochondrial fission (Yeh et al., 2020) (Figure 8). Therefore, the inclusion of Chinese olives in the diet of diabetic patients could be beneficial in controlling blood glucose.

### 4.5 Anti-inflammatory effect

Inflammation is a common stress response induced by many chronic diseases such as obesity, hypertension, atherosclerosis, cancer, and others. Numerous studies have demonstrated that Chinese olive has anti-inflammatory activity. Nine pairs of new enantiomers of benzofuran neolignans (1a/1b−9a/9b) isolated from the 70% methanol fraction of Chinese olive exhibited anti-inflammatory properties, inhibiting NO production in the LPS-induced RAW 264.7 macrophages. Among them, neolignans 1b, 2b, 6a, 7a, 8a, 8b, and 9a had more potent inhibitory effects with IC_50_ values ranging from 6.0 ± 1.7 to 23.5 ± 3.5 μM, all significantly lower compared to the positive control indomethacin (IC_50_ = 25.5 μM). More detailed studies demonstrated that compounds 6a and 6b exerted their anti-inflammatory effects by blocking NF-κB nuclear translocation and reducing the expression of pro-inflammatory mediators COX-2, iNOS, IL-1β, and IL-6 (Li et al., 2022). The anti-inflammatory effect of the ethyl acetate fraction of Chinese olive was also demonstrated. Ethyl acetate fraction had no effect on RAW 264.7 cells’ growth and inhibited the LPS-induced NO production. Further analysis of its active components showed that the anti-inflammatory effects were exerted by bromsitoindoside I, amentoflavone, tetrahydro amentoflavone, and protocatechuic acid (Kuo et al., 2019).

Neuroinflammation is an inflammation type associated with the nerves and is regarded as the main pathogenesis of neurodegenerative diseases. Microglia are the major immune cells in the brain, and their activation is a major feature of neuroinflammation in the central nervous system. Using lipopolysaccharide (LPS) -induced microglia (BV-2) as a model, minocycline was used as a positive control to evaluate the anti-neuroinflammatory activity of Chinese olive. The Chinese olive compounds balanophonin, (7S,8R)-threo-1’-[3′-hydroxy-7-(4-hydroxy-3-methoxyphenyl)-8-hydro-xymethyl-7,8 dihydrobenzofuran] acrylaldehyde, erythro-guaiacylethoxy glycerol-β-O-4′-guaiacyl aldehyde ether and ferulic aldehyde had strong inhibitory activity, reducing NO production, with IC_50_ values 5.26 ± 0.43 μM, 3.02 ± 2.35 μM, 7.38 ± 0.31 μM, and 8.04 ± 2.15 μM, respectively (Zhang et al., 2019). These studies provide scientific support for using Chinese olive as a TCM in treating inflammation-related diseases and demonstrate its potential to be exploited as a natural nutrient and functional food ingredient.

### 4.6 Anti-tumor effect

With the improvement of people’s living standards, cancer incidence is also increasing yearly. Nowadays, although surgery and chemoradiotherapy are still the standard clinical treatment for cancer, the effects of TCMs have been increasingly recognized by both physicians and patients. As research has become more extensive, the effects of Chinese olives in treating malignant tumors have been increasingly revealed. The Chinese olive extract effects on HCT116 (a human colon cancer cell line) and CT26 cells (a mouse colon carcinoma cell line) and the related molecular mechanisms were investigated. Chinese olive extracts, compared with the blank group, could significantly inhibit HCT116 and CT26 cell proliferation and promote cell apoptosis, which was further confirmed by flow cytometry. Inflammation is a key component of malignant tumor progression. NF-κB is a ubiquitous regulator of nuclear transcription factors in eukaryotes, regulating the interaction between inflammation and tumor growth at various levels. Chinese olive extract was shown to have a significant inhibitory effect on LPS-induced NF-κB activation, further inhibiting the downstream expression of COX-2 and iNOS. Western blotting analysis indicated that the Chinese olive extract could effectively reduce the phosphorylation of NF-κB related proteins such as IKK*α*/*β*, IκBα, and p65 and could also inhibit the LPS-induced HCT116 proliferation. Therefore, the Chinese olive extract has an inhibitory activity towards colorectal cancer progression by blocking NF-κB signal transduction and promoting cell apoptosis (Hsieh et al., 2016).

### 4.7 Effect on gut microbiota

Gut microbiota plays a vital role in maintaining immune and metabolic homeostasis and assists physiological functions such as enhancing gut integrity, energy absorption, and resistance against pathogenic microbiota. When the gut microbiota composition and species abundance change, it can result in changes in the body’s physiology and pathological conditions. The effect of Chinese olive extract on the gut microbiota composition of mice was explored using Kun Ming (KM) mice fed with HFD as a model system. After continuous gavage of mice for 4 weeks, mice feces were collected. The gut microbiota composition and diversity were evaluated using high-throughput DNA sequencing. Compared to the normal control group, the relative abundance of Firmicutes increased, whereas that of Bacteroidetes decreased in all HFD-fed mice. The relative abundance of *Allobaculum* genus increased, while the Bacteroidakes-S24-7 genus relative abundance was reduced. Compared with the normal control and model control group, *Verrucomicrobia* and *Akkermansia* relative abundance was significantly increased. In conclusion, the Chinese olive extract was shown to regulate gut microbiota diversity and richness affecting the gut microbiota community structure. It can increase the relative abundance of probiotics while decreasing harmful bacteria (Zhang et al., 2018). The above experiments provide evidence that daily consumption of Chinese olives can regulate gut microbiota with health-promoting effects.

## 5 Practical applications

With the gradual increase of research on Chinese olive, its application is becoming more and more widespread, and related patents are becoming increasingly abundant. The LENS website (https://www.lens.org/) treats comprehensive academic and patent knowledge as a public product, providing information for scientific and technological problem-solving. Using “Chinese olive (*Canarium album* Rauesch.)” as the search term, approximately 800 patents can be found on this website, involving countries such as China, the United States, Korea, India, etc. These patents mainly involve traditional medicine, food science, pharmacology, polysaccharides, molecular biology, etc. We summarize the food, cosmetics, and medicine fields that have been extensively studied, in order to provide research ideas or industrial application directions for relevant researchers.

### 5.1 Applications in the field of food

Chinese olive is nutrient-rich and is a delicious and naturally healthy food. Chinese olives have been consumed for a long time as a fruit having a sweet and sour taste and a lasting aftertaste (Lai et al., 2022). At the same time, it is also commonly used for food seasoning. It can be cooked with fish, pork, beef, etc., to improve the taste of dishes. Because Chinese olives contain many phenolic acids, amino acids, and other compounds, they provide multiple taste stimuli and rich taste when they are included in the cooking. Different ways of making food bring different sensory experiences. Chinese olives can be used to make tea, and their beverage has a light green color, a smooth taste, and effective dissolution of active ingredients, which is beneficial for health. Therefore, it is an ideal fruit tea. Chinese olives are also a natural ingredient for making functional drinks. A patent shows that using Chinese olives as the main raw material and blending with other natural plant ingredients can produce a natural, healthy, and immune enhancing beverage. This drink has a moderate acidity and sweetness, a refreshing taste, and is a natural drink that combines nutritional and health functions. It is very suitable for daily consumption. Like most fruits, Chinese olives can also be used for winemaking, as they are rich in carbohydrates and fermentable sugars. Chinese olives can produce wine with a unique flavor after undergoing yeast fermentation (Huang et al., 2022).

With the advancements in Chinese olive fundamental research, the identification and extraction of functional components and the development of healthy food products are increasing (Yeh et al., 2023). Products differing in nutrition, palatability, and functional properties have been developed, including Chinese olive dried fruit, fresh juice extract, a chewable tablet, oral liquid, and many others. With Chinese olive as the raw material, various preserved products have been developed with good taste that is deeply supported by consumers. It is an ideal raw material for snacks for leisure time, such as Chinese olive preserves and Chinese olive candies. Chinese olive chewable tablets and an olive oral liquid largely retain the nutrients and functional components of olives while also being easy to carry and consume. In addition to the above applications, Chinese olives can be used as nutritional additives in biscuits, bread, porridge, juice, and other products (Lin et al., 2017). Due to the presence of flavonoids and phenolic acids, the Chinese olive extract has good antioxidant properties; thus, it can be used as a food preservative.

### 5.2 Applications in the field of cosmetics

Chinese olive has broad application potential in the daily chemical industry. It shows a satisfactory application prospect in skin care products. Products containing Chinese olive or its extracts span cosmetics, wash supplies, oral hygiene products, fragrances, etc.

Flavonoids, phenols, amino acids, VC, and other Chinese olive active ingredients can be orally absorbed when ingested but can also be absorbed through the skin, having a high utilization value in the beauty and skincare industry and products (Nagula and Wairkar, 2019; Farjadmand et al., 2021; An et al., 2023). As a raw material for cosmetic products, Chinese olive can reduce skin irritation and improve and solve skin problems between deep skin care and non-etiological skin problems. Cleansers, bath creams, and soaps containing Chinese olive extract bioactive ingredients can clean the skin. The developed facial creams, eye creams, toners, and essences are ideal skin care products. At the same time, these products have moisturizing, wrinkle removal, whitening, antioxidant, and anti-aging properties. These olive extracts can also be added as a raw material to beauty products, such as eye shadow, powder blusher, lipstick, concealer, etc., to reduce sensitive skin irritation to chemical ingredients. Moreover, it is also an important raw material and additive in certain functional skin care products. The skin mask containing Chinese olive extract had a better contact with the skin surface cells at the time of use. Its antioxidant components can effectively inhibit the oxidative activities of free radicals, reduce the oxidative effects of free radicals on skin cells, and exert an anti-aging effect (Guo et al., 2008; Wang et al., 2021). Sunscreens containing Chinese olive extracts can effectively block and absorb ultraviolet light to prevent the skin from tanning or sunburn and can also play a role in skin repair after Sun exposure. Lipsticks containing Chinese olive oil can moisturize and protect the lips as they are natural edible products, they are safer and non-toxic. In addition, many toothpaste patents containing Chinese olive have been approved by China National Intellectual Property Administration (CNIPA). Adding effective ingredients of TCM to the toothpaste to better prevent and treat common oral diseases such as inflammation, bad breath, and gum swelling and pain. At present, these products are available for purchase in major shopping malls and supermarkets. With the ongoing comprehensive research, the Chinese olive application values started to be realized, and more products containing it or their extracts will be used in people’s daily life.

### 5.3 Applications in the field of medicine

Chinese olive is included in the affinal drug and diet and the Chinese Pharmacopoeia, and it plays an important role in the medical field of medicine as a medicinal plant and TCM (The Commission of Chinese Pharmacopoeia, 2020). As a natural medicinal plant, fresh food is the most direct way to be used as medicine. When there is a cough or a sore throat, 5 fresh fruits can be consumed. After heavy alcohol drinking, 10 fresh fruits can be consumed. Symptoms of diarrhea and vomiting can be reduced by boiling water with Chinese olive.

As a TCM, Chinese olive is used in formulations with other TCMs and then decocted to form a decoction, which is also a common form of its medicinal use. Chinese olives can be decocted with some TCMs that have anti-inflammatory, antibacterial, and antiviral effects to make the Qingguo-Jiegeng decoction for treating chronic pharyngitis, and with TCMs that promote digestive function and protect the gastric mucosa to make the Qingguo-Shanzha decoction to treat chronic atrophic gastritis. It can also be applied externally on the gums to treat gum ulceration and externally on the skin’s diseased parts to treat eczema.

In addition, Chinese olives can be used as the main raw ingredient to produce Chinese patented medicine with other medicinal materials, facilitating the commercial use of medical commodities to treat common diseases. Qingguo Wan is a patented Chinese medicine included in the Chinese Pharmacopoeia. It is composed of Chinese olive and seven other medicinal materials. It is administered in a water-honeyed or honeyed pill form to treat sore throat and dry cough (Liu et al., 2021). Chinese olive is also the main ingredient of the Chinese patented medicines Qingyan Runhou pian, Ganjie Bingmei pian, and Qingguo keli. They are prepared in various forms, such as tablets, capsules, and granules, to enhance their efficacy and facilitate ingestion. They have commonly used drugs for the treatment of voice hoarseness and pharyngitis. Thus, Chinese olive, its extracts, and its bioactive components have good applications and potential in developing new drugs.

## 6 Conclusion and prospects

Delicious taste, rich nutritional value, and health benefits have become the definition of higher standards of ideal food products. As an edible fruit with high nutritional and medicinal value, the Chinese olive has been increasingly accepted and loved by consumers, meeting people’s needs for healthy food. In recent years, the Chinese olive has attracted increased interest from nutrition and food researchers and natural plant and herbal medicine research institutes, and its value is currently exploited and applied. Chinese olives contain numerous nutrients, such as essential and non-essential amino acids, various fatty acids, organic acids, vitamins, microelements, high-quality dietary fiber, and phytochemical compounds with diverse structures, such as phenolic acids, flavonoids, phenylpropanoids, and other bioactive small molecules. They potentially contribute to the Chinese olive various health benefits, including antioxidant, anti-inflammatory, anti-cancer, hepatoprotective effect, regulation of gut microbiota, and certain pharmacological activities such as anti-*Helicobacter pylori*, anti-influenza, and anti-diabetes properties. Chinese olives have gained increasing interest for their great development potential as an ingredient in healthy foods, nutritional supplements, and natural medicines.

As a fruit, the Chinese olive contains numerous nutrients, which the public and the research community have recognized. Meanwhile, its phytochemical composition has also attracted much attention. Eighteen novel neolignanes phytochemical compounds have been recently isolated from Chinese olives. Their structures have unique characteristics that have not been observed before. Nine pairs of spatial enantiomers have benzofuran structures. At the same time, they exhibit very high anti-inflammatory properties. These findings expand the understanding of Chinese olive anti-inflammatory bioactive components. In addition, research on Chinese olive phytochemistry has explored the effects of different extraction solvents from the perspective of solution polarity and solubility. Previously, the most common extraction solvents used were water, methanol, ethanol, etc. Currently, studies have been conducted on Chinese olive extraction with small polar chloroform to explore its rarely studied polar range, obtain a series of new compounds that have not been previously obtained, and explore the polar distribution and structural characteristics of Chinese olive chemical components. These findings supplement our knowledge of the phytochemical composition of Chinese olives, provide a basis for the discovery and evaluation of novel bioactive components, and provide a reference for identifying and elucidating the underlying biosynthetic pathways. The identification of bioactive markers and biogenic synthesis pathways can guide the optimization of Chinese olive cultivation, determine its picking and conservation, and evaluate its quality. Although Chinese olive have multiple health benefits, their active ingredients are limited and it is difficult to treat diseases solely through daily intake. Nevertheless, consuming Chinese olive is beneficial for physical health and can be used to prevent diseases.

Numerous studies have confirmed the potential benefits of Chinese olives to the human body. As Chinese olives have been used in our diets for a long time, we have accumulated evidence of their health benefits. Modern pharmacological experiments have also provided the scientific basis for *in vitro* and *in vivo* research using cell culture and animal models. However, many pharmacological studies on Chinese olive evaluate its biological activity based on crude extracts or semi-purified components, with only a few directly evaluating specific active components. Although it is extremely important to clarify the biological activity of the crude extract, it is also of great significance to guide the manufacturing of special functional products from the Chinese olive extract. However, identifying the specific biological activity of the Chinese olive’s chemical composition is the key to developing further and fully utilizing its value and realizing its potential applications. The structure-activity relationship of the Chinese olive chemical components is not clear at present, which is an important research topic for the future. Future research directions should focus on advanced analytical and pharmacological tools to isolate and characterize specific bioactive components and to establish their mode of action and structure-activity relationships. More emphasis should be placed on synthetic biology approaches, which may provide an exciting scope for the complete or partial reconstruction of its metabolic biosynthetic pathways.

Additionally, modern pharmacological studies have demonstrated that Chinese olives have unique advantages in combating *H. pylori* and influenza A virus. It is estimated that *H. pylori* infection affects about 50% of the world’s population, causing gastrointestinal diseases and even gastric cancer. It is the only microbial species known to survive in the human stomach, which leads to the lack of bioactive ingredients and precursor compounds for drug research and development. It is difficult to eradicate *H. pylori* because of the widespread use of antibiotics, which leads to drug resistance. Preliminary experiments proved that Chinese olives exhibit inhibitory effects towards *H. pylori* by inhibiting growth, disrupting bacterial structure, and downregulating virulence factors expression. These findings provide leads for further exploration of new treatment schemes and new product development directions. Further studies should be carried out to clarify the chemical components with activity against *H. pylori*. To study the basis of the anti-*Helicobacter pylori* properties, extraction, and purification of monomers with defined structure should be carried out, along with chemical modifications, to select the more effective target molecules. Subsequently, *in vivo*, experimental verification should be performed to understand its mechanism of action in the human body. This will help promote the development of safer and more effective functional food or effective drugs. In addition, Chinese olives possess significant biological activity against influenza. Research on its mechanism of inhibiting the influenza virus has shown that Chinese olive constituents act as neuraminidase and PB2 inhibitor to block the transmission and replication of the influenza virus. Thus, they can be further exploited and developed into a natural source of anti-influenza drugs. The chemical plant components from Chinese olives show a variety of biological activities. Their advantages are that they are derived from natural plants, are safe and non-toxic, and can be developed into various products.

**FIGURE 9:**
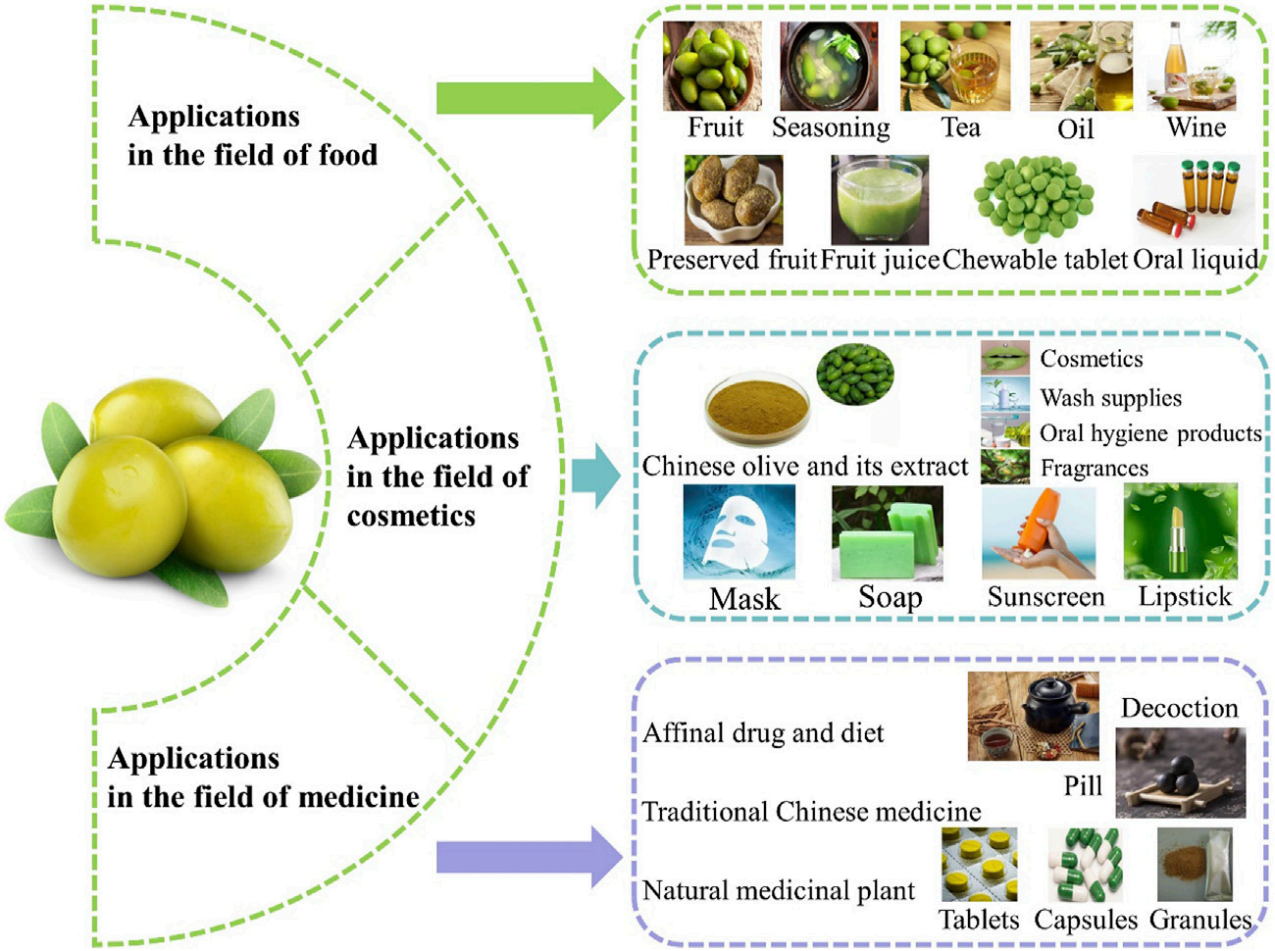
Practical and potential applications of Chinese olive. (Images from network and public sources).

At this stage, a large-scale and mature whole industry chain has been formed around Chinese olive, which is used as fresh fruit and processed into various products to meet different needs (Figure 9). Most of the Chinese olive processed products are at the primary processing level, using simple processes and a low utilization rate of effective ingredients. In the future, modern and novel scientific and technological methods can be applied for the in-depth research, development, and manufacturing of processed Chinese olives to improve their nutritional value and economic attributes. In particular, consumers’ pursuit of a healthy diet and understanding of functional food has become a significant trend.

As an “affinal drug and diet” plant, Chinese olive has clear advantages and a huge potential to be developed into a nutritional supplement and functional food. As its research continuously increases, a higher number of products reflecting its nutritional value will appear in the market. Furthermore, during Chinese olive processing and production, the by-product fruit residue is often discarded as it is considered to be worthless. Reintroducing added-value products obtained from food waste materials into the economic cycle is crucial for the food industry. Numerous bioactive components are present in Chinese olive, which can be obtained by establishing easy-to-set-up and operating extraction methods and optimizing appropriate purification processes. These obtained natural components usually have a very high added value. On the one hand, the reasonable utilization of Chinese olive residues can reduce environmental pollution and waste management expenses. On the other hand, it can use the existing plant resources efficiently so that different Chinese olive industry chain components can generate value. Although the Chinese olive industry is developing continuously, the Chinese olive varieties are usually significantly different and have their regional characteristics in different areas. Data on their genetic structure and phytochemical composition are still imprecise, and many problems need to be solved with comprehensive approaches. Overall, the Chinese olive is a highly nutritious food and a source of bioactive ingredients in empirical medicine. More focus on its research and development should be placed due to its rich nutritional value, broad biological activity, and potential development potential in the field of nutrition and food.
